# A targeted amplicon sequencing panel to simultaneously identify mosquito species and *Plasmodium* presence across the entire *Anopheles* genus

**DOI:** 10.1111/1755-0998.13436

**Published:** 2021-06-19

**Authors:** Alex Makunin, Petra Korlević, Naomi Park, Scott Goodwin, Robert M. Waterhouse, Katharina von Wyschetzki, Christopher G. Jacob, Robert Davies, Dominic Kwiatkowski, Brandyce St. Laurent, Diego Ayala, Mara K. N. Lawniczak

**Affiliations:** 1 Wellcome Sanger Institute, Hinxton, Cambridge, UK; 2 European Molecular Biology Laboratory, European Bioinformatics Institute, Hinxton, Cambridge, UK; 3 Department of Ecology and Evolution, University of Lausanne, and Swiss Institute of Bioinformatics, Lausanne, Switzerland; 4 MIVEGEC, Univ. Montpellier, CNRS, IRD, Montpellier, France; 5 CIRMF, Franceville, Gabon

**Keywords:** high-throughput sequencing, malaria, population genetics, species identification, vector surveillance

## Abstract

*Anopheles* is a diverse genus of mosquitoes comprising over 500 described species, including all known human malaria vectors. While a limited number of key vector species have been studied in detail, the goal of malaria elimination calls for surveillance of all potential vector species. Here, we develop a multilocus amplicon sequencing approach that targets 62 highly variable loci in the *Anopheles* genome and two conserved loci in the *Plasmodium* mitochondrion, simultaneously revealing both the mosquito species and whether that mosquito carries malaria parasites. We also develop a cheap, nondestructive, and high-throughput DNA extraction workflow that provides template DNA from single mosquitoes for the multiplex PCR, which means specimens producing unexpected results can be returned to for morphological examination. Over 1000 individual mosquitoes can be sequenced in a single MiSeq run, and we demonstrate the panel’s power to assign species identity using sequencing data for 40 species from Africa, Southeast Asia, and South America. We also show that the approach can be used to resolve geographic population structure within *An*. *gambiae* and *An*. *coluzzii* populations, as the population structure determined based on these 62 loci from over 1000 mosquitoes closely mirrors that revealed through whole genome sequencing. The end-to-end approach is quick, inexpensive, robust, and accurate, which makes it a promising technique for very large-scale mosquito genetic surveillance and vector control.

## 1 Introduction

The genus *Anopheles* includes about 500 species that are categorised into seven subgenera and further divided into sections, series, groups, and species complexes (Harbach, 2013a; Harbach & Kitching, 2005, 2016). Major malaria vectors are distributed among four of these subgenera: *Nyssorhynchus, Anopheles, Cellia*, and *Kerteszia*. Important vector species are often found within species complexes where ongoing speciation and hybridization involves both vector and nonvector species, for example, species within the *Gambiae* complex (Barrón et al., 2019; Crawford et al., 2015; Fontaine et al., 2015; Thawornwattana et al., 2018), the *Funestus* group (Coetzee & Koekemoer, 2013) in Africa, and *Leucosphyrus* group containing the *Dirus* complex (Walton et al., 1999) in Southeast Asia. This tendency for many *Anopheles* species to have permeable species boundaries underscores the need for a much more nuanced approach to determining the species that a sample belongs to than single marker methods allow.

The typical workflow for species identification in *Anopheles* usually starts with morphological identification to classify the species complex or group using the key for the appropriate geographical region (Gillies & Coetzee, 1987; Gillies & De Meillon, 1968; Irish et al., 2020; Rattanarithikul & Panthusiri, 1994). Assignment to precise species within these groups or complexes is frequently not possible using morphology alone. Therefore, the most commonly used approach to discriminate closely related species is based on PCR targeting the highly variable internal transcribed spacer (ITS2) in the nuclear rRNA gene cluster. Using cocktails of universal plus species-specific primers it is possible to generate PCR products of distinct lengths (Cohuet et al., 2003; Scott et al., 1993). For the most closely related species, additional steps can be required. For example, the ITS2 sequences of *An*. *gambiae* and *An*. *coluzzii* are discriminated using a restriction enzyme site with known single-nucleotide differences between the species (Fanello et al., 2002), although assays that do not require further digestion also exist (Wilkins et al., 2006). PCR and gel-based methods can fail due to mutations in primer or restriction sites, or more drastic morphological misidentification to the wrong complex/group, and single marker assays can also give misleading answers due to introgression, a common feature among *Anopheles* species. Furthermore, methods that lead to conclusions based on gel bands can generate both false negatives in the case of PCR failures as well as false positives for species outside of the studied group (Erlank et al., 2018). More complex assays targeting multiple genomic sites such as microsatellites partially overcome these issues and provide resolution for population structure, but only for a few restricted groups of species where much more work has been invested (Lanzaro et al., 1995; Rongnoparut et al., 1996; Santolamazza et al., 2008; Wang-Sattler et al., 2007).

Several molecular species identification approaches using sequence data are applicable to any member of the genus. These use conserved PCR primers and require Sanger sequencing to reconstruct the sequence of the variable inserts. The most frequently used markers are ITS2 and mitochondrial cytochrome oxidase c subunit I (COI) (Sallum et al., 2002; Stevenson et al., 2012; St Laurent et al., 2016) and II (COII) (Ayala et al., 2019; Rahola et al., 2014). However, their power to resolve closely related species is limited (Beebe, 2018; Krzywinski & Besansky, 2003).

Clearly, single markers are problematic for understanding the true landscape of species diversity in *Anopheles*. Targeted amplicon sequencing approaches can be used across multiple regions of the genome to give a more comprehensive view into species assignment and appropriate targets can be selected when comparative genomics data are available (Lemmon & Lemmon, 2012). Such approaches can be used to reconstruct species trees (Barrow et al., 2014) and to improve species delineation and hybrid detection (O’Neill et al., 2013; Wielstra et al., 2014). Technological advancements allow for hundreds of amplicons to be quickly designed, multiplexed in a single reaction, and efficiently used at the required taxonomic scale (Dupuis et al., 2018). A similar approach can now be applied to *Anopheles* mosquitoes, thanks to the growing body of reference genomes across the genus (Giraldo-Calderón et al., 2015; Neafsey et al., 2015; Ruzzante et al., 2019) as well as large numbers of sequenced individuals within some species (Anopheles gambiae 1000 Genomes Consortium, 2017).

Here, we present a multilocus amplicon panel that can be applied to any *Anopheles* individual without prior information on the species complex or group of which it is a member. We also develop a cheap, nondestructive, and high-throughput DNA extraction workflow that provides template DNA from single mosquitoes for the multiplex PCR, which means specimens producing unexpected results can be returned to for morphological examination. Barcoded amplicons from over a thousand individual mosquitoes can be combined into an Illumina library pool that, when run on a single Illumina MiSeq lane, simultaneously reveals species identity, and *Plasmodium* presence status, and gives a window into population structure for each species. We hope that the amplicon panel we report here will be broadly used in vector control and surveillance given it is a leap forward in producing higher throughput, cheaper, more accurate, and more informative data in comparison to current species identification techniques. Through application of this panel to more samples from more species, we believe it will refine our understanding of species diversity, population structure, and malaria transmission across the genus.

## 2 Materials and Methods

### 2.1 Whole-genome marker selection

The initial selection of phylogenetically informative markers was based on the whole-genome alignment of 21 genomes from 19 species representing three *Anopheles* subgenera: *Anopheles*, *Cellia*, and *Nyssorhynchus* (Neafsey et al., 2015). The whole-genome alignment strategy is detailed in Neafsey et al., (2015), briefly: the MULTIZ feature of the Threaded-Blockset Aligner suite of tools (Blanchette et al., 2004) was used to progressively combine all-against-all pairwise alignments guided by the species phylogeny. We extracted potential amplicon regions using the following criteria: at least 10 species had alignments available for the region; the region was flanked by at least 40 bp of conserved sequence (maximum of 10% substitutions and 10% indels); the amplicon length could vary between 150 and 280 bp. A total of 940 regions met these criteria and were examined further.

The obtained regions were annotated using the AgamP3 (differing from most recent assembly AgamP4 by the lack of mitochondrial genome only) gene set 8 from VectorBase (https://www.vectorbase.org/). Most regions fell inside exons, sometimes overlapping UTRs or spanning short introns. Regions spanning introns demonstrated high levels of sequence variability even on the level of populations, suggesting potential to reveal more fine-grained population-scale variation. Additional annotations using the repeat library (RepeatMasker, Dust, TRF - v.1.00 at VectorBase) were used to remove regions overlapping with microsatellites and transposable elements. Next, we evaluated sequence variation of the potential amplicon regions and excluded those where fewer than 10 distinct sequences were found for the 10 to 21 genomes.

To estimate higher level conservation, the AgamP3 sequences of the regions were aligned against outgroup dipteran genomes: *Culex pipiens* CpipJ2, *Aedes aegypti* AaegL5, and *Drosophila melanogaster* DmelBDGP6 using blastn v.2.7.1. We also aligned those regions to AgamP3 to identify and remove markers that were duplicated. The final design was tested on *Culex* and *Drosophila* samples and showed some phylogenetic signal for both, allowing discrimination from *Anopheles* samples (see Supporting Information Methods section “Panel applicability on outgroup species” for details). After the above filtering steps, a total of 591 potential amplicons remained.

### 2.2 Panel design

Primer design was performed using an in-house modified version of mprimer (Qu et al., 2012; Shen et al., 2010). The program modifications vastly speed up the process of multiplex PCR design without any effect on the resultant primer design output itself. The initial list of 591 target sites was sorted to ensure that amplicons deemed highly informative were prioritized. Highly informative amplicons spanned three categories including those that were highly variable and supported discrimination across most lineages (*n* = 22 available); those that were highly conserved, that is, retained alignments in at least one of the three outgroup species (*n* = 27 available); and those located on the *An*. *gambiae* X chromosome, while also being found in most other species (*n* = 6 available). The remaining 536 amplicons were sequentially added for design prioritizing those that were likely to reveal the highest number of distinct sequences across species, and then by coordinates in the AgamP3 genome to spread targets across all chromosome arms.

We also incorporated several different conservation requirements for the primers themselves (rather than the full amplicon sequence) to ensure the greatest chance of successful primer binding across highly divergent species. The sequence for each amplicon target region was provided as an input FASTA file for each of the following: masking at all sites that differed between species, masking at any site that had a diverged site in two or more of the reference genomes, and no masking. Primer pairs were selected for each target region using these three masks sequentially—first allowing no variants in primers, then allowing for variants in a single aligned genome, and finally fully relaxing these restrictions. For each input file, mprimer was run using the following parameters: primer min_gc 20, primer opt_gc 50, primer max_gc 80, primer min_tm 50, primer opt_tm 58, primer max_tm 68, product_min_size 190 bp, product_ max_size 250 bp, penalty allowance 10. In the final design, degenerate bases were used in 23 primers in cases of polymorphisms found in two or more genomes.

**Figure 1:**
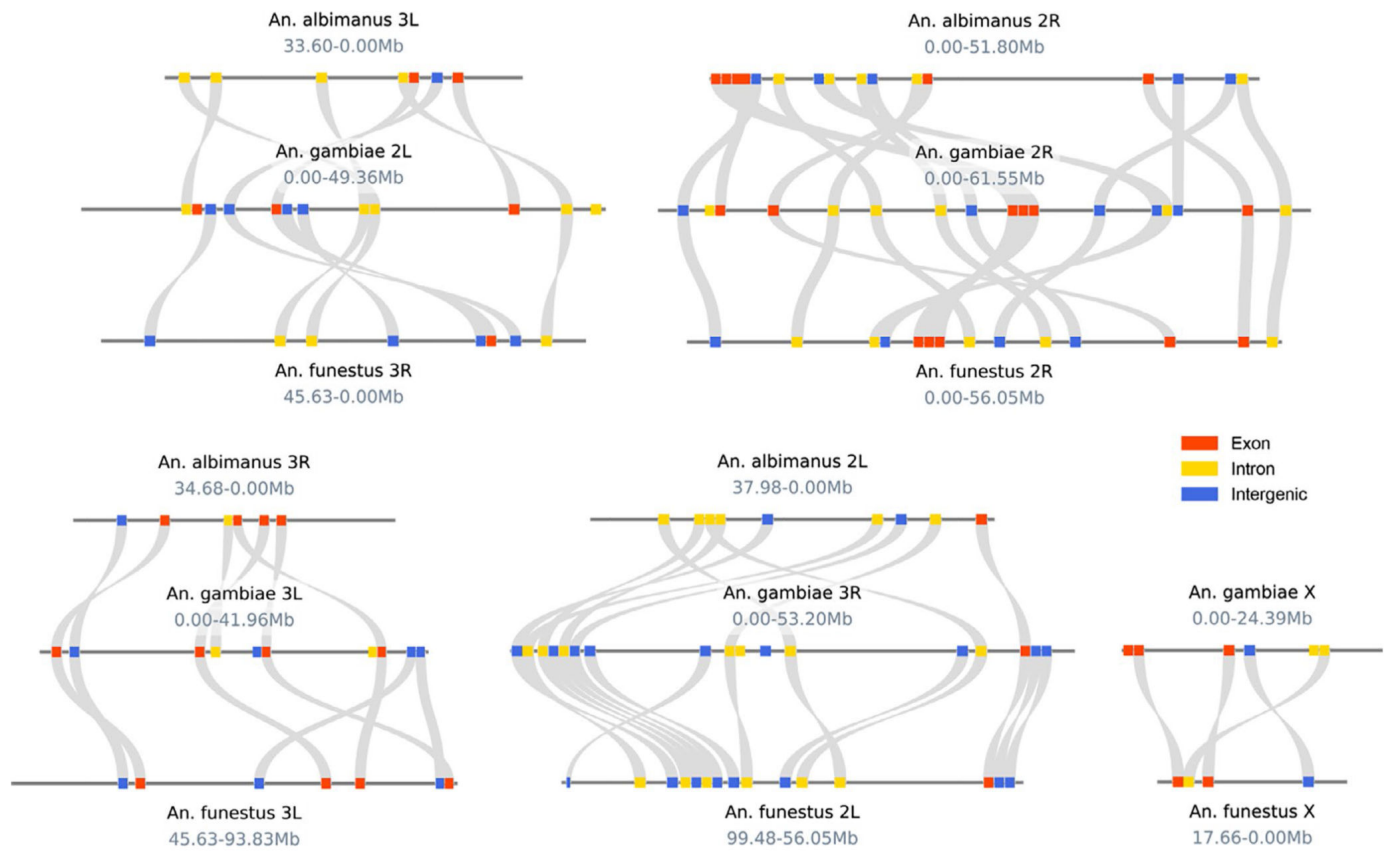
Positions of 62 amplicons in three *Anopheles* genomes: *An*. *albimanus* (top), *An*. *gambiae* (center), and *An*. *funestus* (bottom). Colours indicate marker types based on the AgamP3 gene set: exonic (red), intronic (yellow), or intergenic (blue). The *An*. *albimanus* X chromosome is not represented due to a lack of amplicon homologues. No translocations between chromosome arms were observed

A total of 62 primer pairs were picked. For the highly informative categories listed above, we retained four (of 6) X-linked, 14 (of 22) variable, and nine (of 27) conserved amplicons. In the *An*. *gambiae* genome, the resulting 62 amplicons are distributed across the autosomes and the X chromosome, with 17 that fall within exons, 22 that span introns, and 23 that are intergenic (Figure 1, Supporting Information File S1). Comparative visualisation of amplicon locations in multiple genomes was generated in jcvi v.0.9.13 (Tang et al., 2015).

In order to assess *Plasmodium* infection status and species identity, two additional primer pairs were selected based on the mitochondrial genome alignments of five *Plasmodium* species that infect humans: *P*. *falciparum*, *P*. *vivax*, *P*. *malariae*, *P*. *ovale*, and *P*. *knowlesi*. Both amplicons overlapped several ribosomal RNA fragment genes (Feagin et al., 2012): for P1—RNA9 (forward primer), RNA17, RNA23 t, large subunit fragment C, RNA24 t, RNA25 t, large subunit fragment G (reverse primer); for P2—small subunit fragment E (forward primer), RNA2, RNA21, RNA26 t, RNA3 (reverse primer). We tested the ability of the panel to amplify and discriminate *P*. *falciparum* and *P*. *berghei* using pure parasite DNA as well as laboratory-infected mosquitoes. blast results post-sequencing confirm parasite species discrimination is accurate (data not shown).

Thus, the final version of the multiplex PCR panel included 64 pairs of primers, with 62 targeting the *Anopheles* nuclear genome and two targeting the *Plasmodium* mitochondrial genome. Mitochondrial markers were selected for *Plasmodium* detection because the copy number of *Plasmodium* mitochondria inside a mosquito is likely to be much higher than for the *Plasmodium* nuclear genome and should increase the sensitivity of parasite detection in what is likely to be majority mosquito DNA.

To enable sample barcoding (described below) coupled with Illumina library preparation, a common Illumina adapter sequence motif was added to each primer sequence: 5′-ACACTCTTTCCCTA CACGACGCTCTTCCGATCT-[amplicon-specific forward]-3′, 5′-TCG GCATTCCTGCTGAACCGCTCTTCCGATCT-[amplicon-specific reverse]-3′, as well as a 2′-*O*-Methyl base exchanged for the penultimate DNA base, used in order to minimise potential primer dimers (McKerrell et al., 2015).

### 2.3 Mosquito samples

We evaluated the performance of the panel to amplify target regions and resolve species relationships by testing the panel on DNA from many previously identified species originating from three continents. From Africa, we included 89 specimens representing 24 species of wild-caught mosquitoes from Gabon that were identified morphologically with further PCR-based species attribution where possible (Ayala et al., 2019). From Southeast Asia, we included 43 specimens representing 13 species of wild-caught mosquitoes from Cambodia that were identified to species level using ITS2 sequencing (Beebe & Saul, 1995). Finally, we obtained a single sample from three species of mosquitoes from Brazil, South America, which were identified morphologically. As a reference for species names, we used the NCBI Taxonomy database with some updates from the Mosquito Taxonomic Inventory (Harbach, 2013b). The details on each specimen together with its storage and extraction methods are summarised in Supporting Information File S2 tab “sample metadata”.

In addition to sequencing wild caught specimens, we also generated the sequences that would have theoretically been amplified using these 62 primer pairs from each of the available 28 genome assemblies comprising 23 *Anopheles* species (Supporting Information File S2 tab “sample metadata”). To do this, we used the seekdeep v.2.6.4 (Hathaway et al., 2018) genTargetInfoFromGenomes command, which looks for primer matches in the genome and returns all of the potential amplification products.

Additional mosquito samples were used to develop and validate nondestructive DNA extraction approaches, as well as to test the sensitivity of the *Plasmodium* primers to detect parasites in infected mosquitoes (both processes described in Supporting Information Methods). For DNA extraction tests, we used laboratory-reared samples of the *An*. *coluzzii* Ngousso strain and wild-caught samples of *An*. *funestus* from Palmeira, Mozambique. For *Plasmodium* primer rebalancing and qPCR validation, we used laboratory-reared *An*. *stephensi* mosquitoes infected via membrane-feeding assays with *P*. *falciparum* gametocyte culture. These infected mosquitoes were sampled at various time points after feeding—0 h, 24 h, 3 days, 8–9 days, and 13–14 days, and unfed controls were also included (more details in Supporting Information Methods). Additionally, DNA from uninfected laboratory-reared *An*. *coluzzii* or *An*. *stephensi* was mixed with a dilution series of separately extracted *P*. *falciparum* 3D7 DNA. Two additional samples of *An*. *stephensi* infected with *P*. *berghei* were included to ensure that *Plasmodium* primers were operational on other parasite species. For outgroup performance testing we used wild-caught *Culicinae* samples from the UK as well as laboratory-reared *Drosophila melanogaster*.

### 2.4 DNA extraction, amplification, and sequencing

#### 2.4.1 Optimised DNA extraction

We have developed a low cost and rapid DNA extraction procedure that relies on a custom made lysis buffer (further referred to as ‘lysis buffer C’, which contains 200 mM Tris pH 8.0, 25 mM EDTA pH 8.0, 0.4 mg/ml Proteinase K and 0.05% Tween 20, as modified from (Santos et al., 2018)) without any need for DNA purification prior to multiplex PCR (see Supporting Information Methods section “DNA retrieval testing”, Figures S1–S6 and Table S1 for further information on optimisation experiments). The final optimised DNA extraction procedure is as follows. Fully submerge individual fresh or desiccated mosquitoes in 60 μl of lysis buffer per well of a 96-well microtiter plate. If samples were previously stored in ethanol, remove most of the volume with a pipette and ensure all of the ethanol is fully evaporated by drying samples in a 37°C incubator until all samples are dry. Once samples are submerged in lysis buffer C, incubate the plate overnight up to 24 h at 56°C. After incubation transfer as much as possible of the 60 μl of lysate to a new plate leaving the mosquito samples behind in the original plate (these can have ethanol added or be air dried for long term storage; we recommend resuspending and storing the mosquito samples at 4°C in 150 μl 100% ethanol). The concentrated lysate can be stored as is or purified and used for whole genome sequencing if warranted (N.B. we have also successfully sequenced whole genomes from samples extracted this way without purification prior to DNA shearing and Illumina shotgun library preparation). If the only aim for the resulting DNA is to use the amplicon panel described here, then no DNA purification is necessary as the lysate can instead be diluted 10-fold with ultrapure water and used directly in the subsequent multiplex PCR, followed by library preparation and Illumina sequencing. The same 10-fold dilution can also be used to quantify the concentration of double stranded DNA in the dilution using the Quant-iT PicoGreen dsDNA Assay Kit (Invitrogen); however, salts and undigested proteins in the dilution will affect the measurements making these only approximate concentration estimates. This workflow minimizes physical effort, sample preparation time, and cost, which we estimate to be £0.09 per sample excluding plastic consumables (Table S2). The bulk of this cost arises from Proteinase K.

#### 2.4.2 Multiplex PCR with integrated Illumina library preparation

Samples were amplified using a bespoke miniaturised high-throughput amplicon sequencing protocol. This is a highly multiplexed two stage PCR, with the first 64-plex “Target Amplification PCR” employing bipartite primers (as previously described in the “Panel design” section) to capture variable genome sites and their surrounding regions (190–250 bp typical size) as well as incorporate Illumina sequencing motifs, and the second “Sample Barcoding PCR” also employing bipartite primers that target the previously-introduced Illumina motifs and introduce dual-index barcodes and Illumina flowcell adaptor sequences (Figure S6).

##### Target amplification PCR

Reactions were set up using a modified TTP Labtech Mosquito HV (incorporating on-deck cooling) for a 5 μl final volume in a 4titude FrameStar 384-well plate, with 1 μl of purified extract or 1:10 diluted crude lysate as input. Qiagen Multiplex mastermix (P/N 206145) was used based on its previously observed superior performance at the Sanger Institute on different samples in multiplex PCR compared to high-fidelity polymerases (data not shown). No effect of the master mix concentration in the range of 1×–1.25× on amplification efficiency was found for unpurified or purified lysate samples (data not shown). Plates were heat-sealed using a Bio-Rad PX1 heat sealer and peelable foil seals.

In order to ensure that targets were captured with approximately equal efficiency and with minimal off-target amplification in spite of the high degree of multiplexing, we deviated from standard practice in the target amplification PCR in several ways. All target amplification primers feature 2'-*O*-Methyl modifications at the penultimate 3′ base to inhibit primer-dimer formation. The average final primer concentration was 300 pM with the exact primer concentration determined by the independent processes for mosquito (Supplorting Information Methods section “Mosquito primer rebalancing”) and parasite (Supporting Information Methods section *“Plasmodium* primer rebalancing”, Figures S7–S14) amplicons. *Plasmodium* detection efficiency was further tested with qPCR (Supporting Information Methods section “qPCR validation of *Plasmodium* detection”, Figures S15–S16). Final primer concentration multipliers are summarised in Supporting Information File S1. Cycling conditions for the Target Amplification PCR included elongated holds at the annealing temperature without subsequent ramp-up to the extension temperature to permit efficient target capture in spite of the low primer concentration as well as a restricted cycle number to provide just-sufficient PCR product for the second PCR stage to be efficient. Target Amplification PCR cycling conditions are as follows: 95°C for 15 min (enzyme activation); 95°C for 20 s (denaturation), 55°C for 40 min annealing and extension repeated for five cycles; 72°C for 3 min (final extension); 4°C hold (ready for preparation of second PCR stage).

##### Sample barcoding PCR

The second round of PCR introduces dual indexing 8 bp barcodes and Illumina-compatible adaptor sequences to all the amplicons generated in the first PCR by targeting the sequencing-read motifs introduced by the 5′ tails of the target primers in the first stage. Due to the low reaction volumes it is not possible to introduce the second stage primers into the PCR in solution form, therefore instead the entire reaction volume is transferred from the first PCR plate into a second plate that contains 1 pmol of the prealiquoted dried dual-index barcoding primers in each well (therefore giving a final concentration of 200 nM in the PCR), and the reactions are mixed thoroughly to rehydrate and disperse these primers into the reaction. Because the Taq present in the reactions has already been activated, in order to inhibit any off-target product formation during this process the transfer is performed at 4°C using the chilled decks of a modified TTP Labtech Mosquito HV, the new plates are heat sealed in the same manner as previously described and the second PCR is started immediately afterwards with the plates transferred from the chilled deck to the preheated PCR block.

The cycling conditions for the second PCR are as follows: 95°C hold (PCR plate transferred directly from 4°C cooled Mosquito deck onto thermocycler block, then rest of protocol commenced); 95°C for 20 s (denaturation), 62°C for 15 s (annealing), 72°C for 20 s (extension), repeated for 31 cycles; 72°C for 3 min (final extension) followed by 4°C hold. These conditions have been carefully chosen to ensure that the carryover first stage primers no longer significantly participate in the second PCR therefore obviating the need to perform any sort of purification between the first and second PCRs (there is approximately a 1000-fold excess of second stage barcoding primer compared to each individual first stage primer pair, and the second PCR annealing times are insufficient to allow efficient amplification by the first stage primers). Furthermore, the cycling conditions achieve plateau by effective exhaustion of the available barcoding primers over a wide range of input, therefore the total yields per PCR are normalised to approximately the same level irrespective of the initial DNA concentration.

#### 2.4.3 Library pooling and sequencing

As the PCR stages have already incorporated Illumina-compatible flowcell adaptor sequences and sequencing read motifs, no further library preparation is required. In addition, as the PCR design achieves a large degree of normalisation across targets and samples, the sequencing library can be made by pooling PCRs in a simple equivolume manner. We do this by removing the plate seal and inverting the PCR plate over a Clickbio VBLOK200 reservoir, followed by gentle centrifugation. An aliquot of the plate pool is then cleaned up with two successive 0.75× volumes of AMPure XP beads, the eluted library pool is checked for expected sizing using an Agilent Bioanalyser with a High Sensitivity chip and then finally the library pool is quantified by qPCR using Kapa’s Illumina library quantification kit. After denaturation and dilution to 16 pM in HT1 buffer as per the Illumina MiSeq System Denature and Dilute Libraries Guide, the library is sequenced on an Illumina MiSeq using a 300 cycle v2 kit using a total of 316 cycles (2 x 150 bp paired end + 2 × 8 cycles for barcode reading).

#### 2.4.4 Single marker Sanger sequencing

Details for species identity validation analysis are outlined in Supporting Information Methods section “Molecular Species ID validation using COI and ITS2 single marker Sanger sequencing” and Figure S17. Briefly, we amplified COI (Folmer et al., 1994) and ITS2 (Beebe & Saul, 1995) products and sequenced those with Sanger technology (Eurofins GATC SupremeRun 96). We performed a homology search in bold (Ratnasingham & Hebert, 2007) and NCBI GenBank. We also aligned the sequences, reconstructed phylogenetic trees and obtained diversity estimates and compared those with amplicon sequencing results.

### 2.5 Sequence data processing

Data processing of the resulting Illumina reads is required to reconstruct the alleles detected at each target amplicon from raw reads. Demultiplexing of samples based on indexing barcodes is done as a part of standard Illumina procedure and results in a pair of fastq files for each sample. The processing of those consists of amplicon demultiplexing based on primer sequences, forward and reverse read merging and reconstruction of alleles that should ignore the erroneous sequences resulting from off-target amplification, PCR, or sequencing errors. Thus, balancing between sensitivity (i.e., false negative rate of missing genotype data) and specificity (false positive rate of spurious genotypes) is an important consideration. Here, we implemented and tested two pipelines using different software—seekdeep v.2.6.4 (Hathaway et al., 2018) and dada2 v.1.10.0 (Callahan et al., 2016) (described in detail in Supporting Information Methods section “Sequence data processing pipelines design and benchmarking”, Figure S18 and Table S3). The key performance difference is that seekdeep is more specific and less sensitive compared to dada2, but only if technical replicates resulting from different PCR reactions are provided for each sample (Table S3). We decided to use dada2 for both species identification and population-level analyses in order to avoid the need for replicates and to generate higher amounts of data, albeit with an elevated error rate. There are also alternative methods for amplicon sequence data processing, for example the HaplotypR (Lerch et al., 2017) and PASEC pipelines (Early et al., 2019), which could be explored for use with the amplicon panel described here.

### 2.6 Distance-based species attribution

The data set used here included mosquitoes collected from Africa, Southeast Asia and South America, as well as target sequences extracted from reference genomes—a total of 161 samples belonging to 57 species from four subgenera (*Cellia—119* samples, 42 species; *Anopheles—36* samples, nine species; *Nyssorhynchus—four* samples, four species; *Kerteszia—two* samples, two species).

Amplicon sequences were aligned using mafft v.7.407 (Katoh et al., 2002). Sequence variation statistics were collected using biopython v.1.74. Sequence clustering was performed for each amplicon independently with cd-hit-est v.4.8.1 (Fu et al., 2012), which uses static sequence similarity threshold to delineate clusters. Two sets of thresholds were generated based on within-species diversity in either the sequenced data set and reference genomes or in the continent-wide population sampling of *An*. *gambiae* and *An*. *coluzzii* (1142 individuals from the Ag1000g Phase2 data set). The performance of both sets of thresholds was evaluated by excessive species splits between clusters and multiple species falling into a single cluster. The set of Ag1000g-derived thresholds proved to have higher species resolution with similar level of splits - presumably due to wider species sampling compared to sequenced mosquito data set, so it was used in the downstream analysis (for details, see Supporting Information Methods section “Distance-based species attribution” and Figures S19–S20).

In order to predict the species of each sample based on clustering results, we generated a “species labelling data set” comprising sequences with reliable species predictions. In this data set, we included all predicted amplicon sequences generated from reference genomes as we consider their species and homology predictions to be robust. For the mosquitoes we sequenced here, we introduced a filter to ensure that only consistent data were included in the species labelling reference data set. In doing this, we did not question the original species labels that were given to samples based on morphology and, in some cases, a single molecular marker. Instead, we focused on internal coherence of the sequencing data—each species should be represented by a set of relatively similar sequences across the samples representing that species. We analysed within-species distances across all amplicons and identified eight samples that were too distant from other samples of that species. For one of the species, *An*. *marshallii*, all three samples were excluded due to this problem (due to suspected morphological misidentification or sample labelling mistake). The resulting species labelling data set included 9589 of 10057 sequences available for both reference genomes and sequenced samples, and represented 56 of 57 species.

For the purpose of species identification, the species labelling data set was clustered together with the remaining sequences. Each cluster for each amplicon was labelled with the set of reference data set species whose sequences fell within that cluster (Figure S21). The species prediction procedure for any given sample consists of two steps: (i) for each sequence available for a sample identify the set of species labels for the corresponding cluster; (ii) find the most frequent species label across these sets. For closely related species that share many alleles, boundaries are not discrete. For example, a species label might be assigned as *An*. *coluzzii* because 75 of 80 alleles detected in the sample are found in the “species labelling data set” assigned to *An*. *coluzzii*, yet 70 of those alleles are also found in the set of alleles assigned to *An*. *gambiae*.

The final version of the species tree was reconstructed based on the samples that were included in the species labelling data set only. Amplicon sequences for each sample were split into two pseudo-haplotypes. Haplotype 1 consisted of the most frequent allele, haplotype 2 consisted of the second most frequent allele or of a copy of haplotype 1 for homozygous genotype calls. For each amplicon, sequences for all haplotypes were aligned with MAFFT and maximum likelihood phylogenies were reconstructed with fasttree v.2.1.10 (Price et al., 2010). The species tree was reconstructed using astral v.5.6.3 (Rabiee et al., 2019), tree visualisation and taxonomy manipulations were performed with ete v.3.1.1 (Huerta-Cepas et al., 2016).

### 2.7 Population structure for *An*. *gambiae* and *An*. *coluzzii*

The Ag1000g Phase 2 AR1 data set of biallelic variants for 1142 individuals of *An*. *gambiae* and *An*. *coluzzii* was subsetted to amplicon target sites, that is, primer sequences were excluded from the analysis. Three available reference genomes were also synthetically analysed at the amplicon sites. We also included three *An*. *coluzzii* from Gabon and five *An*. *gambiae* from Gabon that were sequenced using the amplicon panel. For each amplicon, the fasta sequence alignments were converted to genotype matrices using AgamP3 as a reference, then subsetted to Ag1000g Phase 2 AR1 biallelic sites.

The overall population structure for the combined data set including all *An*. *gambiae* and *An*. *coluzzii* samples was visualised with the Uniform Manifold Approximation and Projection (UMAP) dimensionality reduction technique, using the software umap-learn v.0.3.10 (McInnes et al., 2018). For the Ag1000g data set alone, population variation statistics (nucleotide diversity, Watterson theta, Tajima's *D*) and pairwise population Hudson’s *F*_ST_ were estimated as implemented in scikit-allel v.1.2.1.

## 3 Results

### 3.1 Minimally destructive DNA extraction optimisation

Most of the wild caught *Anopheles* DNA extracts we used were generated using common proprietary tissue lysis and DNA extraction kits. However, due to the potential scale of an amplicon based approach in screening thousands of samples, and the likely discovery of new species that will ensue, we developed an alternative approach to simultaneously protect the morphology of samples so that they can be returned to post sequencing, and also to minimise the per sample extraction costs and required labour. We tested a set of nondestructive DNA extraction protocols with custom lysis buffers, and selected a buffer that resulted in good DNA yields and which, when diluted, could be used directly in PCR without further purification. PCR products and amplicon sequencing results generated using this custom DNA extraction approach were comparable in quality with results obtained from samples prepared using commonly used proprietary DNA extraction kits (Supporting Information Methods section “DNA retrieval testing”).

### 3.2 Panel development and optimisation

Using the alignments of 21 genomes of 19 *Anopheles* species spanning 100 million years of evolution (Neafsey et al., 2015), we selected phylogenetically informative regions with conserved flanks to create a new high-throughput sequencing based approach to assign individual mosquitoes to their appropriate species identity. The final design included 62 amplicons distributed across the mosquito genome (Figure 1) and two additional amplicons to detect and determine *Plasmodium* species (Supporting Information File S1).

The optimised sample preparation for sequencing consists of two PCR reactions. In the Target Amplification PCR, 64 primer pairs are used in a single tube on a single sample. Individual primer concentrations were adjusted to ensure even amplification levels across the whole panel using separate procedures for mosquito and parasite amplicons (see Supporting Information File S1 for the optimised concentrations). In the Sample barcoding PCR, dual indexing barcodes are introduced, allowing for multiple samples (up to 1536 using 96 × 16 barcode set) to be sequenced on a single Illumina MiSeq run. The entire panel design was optimised for 150 bp paired-end reads.

In order to ensure that *Plasmodium* detection with the panel is reliable, we used samples of laboratory-infected mosquitoes and serial dilutions of *Plasmodium* DNA to optimise primer concentrations, test various PCR conditions, and validate infection status with a qPCR protocol (Bass et al., 2008; Djouaka et al., 2016) commonly used in vector surveillance (see Supporting Information Methods section “Plasmodium primer rebalancing” for details). The optimal *Plasmodium* primer concentrations in our multiplex PCR were much higher than the average concentrations of mosquito primers (80x for P1 and 10x for P2), with P2 amplifying more efficiently than P1 (Figure S10). Cross-validation showed high concordance between amplicon sequencing and qPCR prediction of infected samples (90% for P1, 97.5% for P2 with the remainder being false-negatives in amplicon sequencing), although absolute infection rate values were not perfectly correlated (Figure S16). Using a dilution series, we estimated the detection limit for amplicon sequencing as <50 fg, or two parasite genomes in the template provided (Figure S7), a sensitivity similar to qPCR (Figure S16). Given the lysate dilution and aliquoting, this corresponds to about 1000 parasite genomes in a mosquito (a single mature oocyst has more than 1000 copies). The signals agree well with the pattern observed in laboratory-infected mosquitoes (Figures S8 and S11): detection rate and read counts are high at days 0–1 (blood feed with gametocytes still present in all samples) and 13–14 (developed oocysts, some samples not infected), while at days 8–9 (earlier oocysts with <1000 genomes) parasite detection rate and read counts drop. We can also use the difference in sensitivity between primers to predict low-level infections—positive in P2, but negative in P1.

Reconstruction of allelic sequences for each amplicon from raw sequencing data consists of demultiplexing, forward and reverse read merging and removal of erroneous sequences resulting from the off-target amplification, PCR or sequencing errors. We implemented and tested data processing pipelines based on seekdeep (Hathaway et al., 2018) and dada2 (Callahan et al., 2016). dada2 was chosen for further work as it performs better in the absence of multiple PCR replicates per sample and yields more data, though with a small increase in error rate (Supporting Information Methods section “Sequence data processing pipelines design and benchmarking”, Table S3).

### 3.3 Panel informativeness

**Figure 2:**
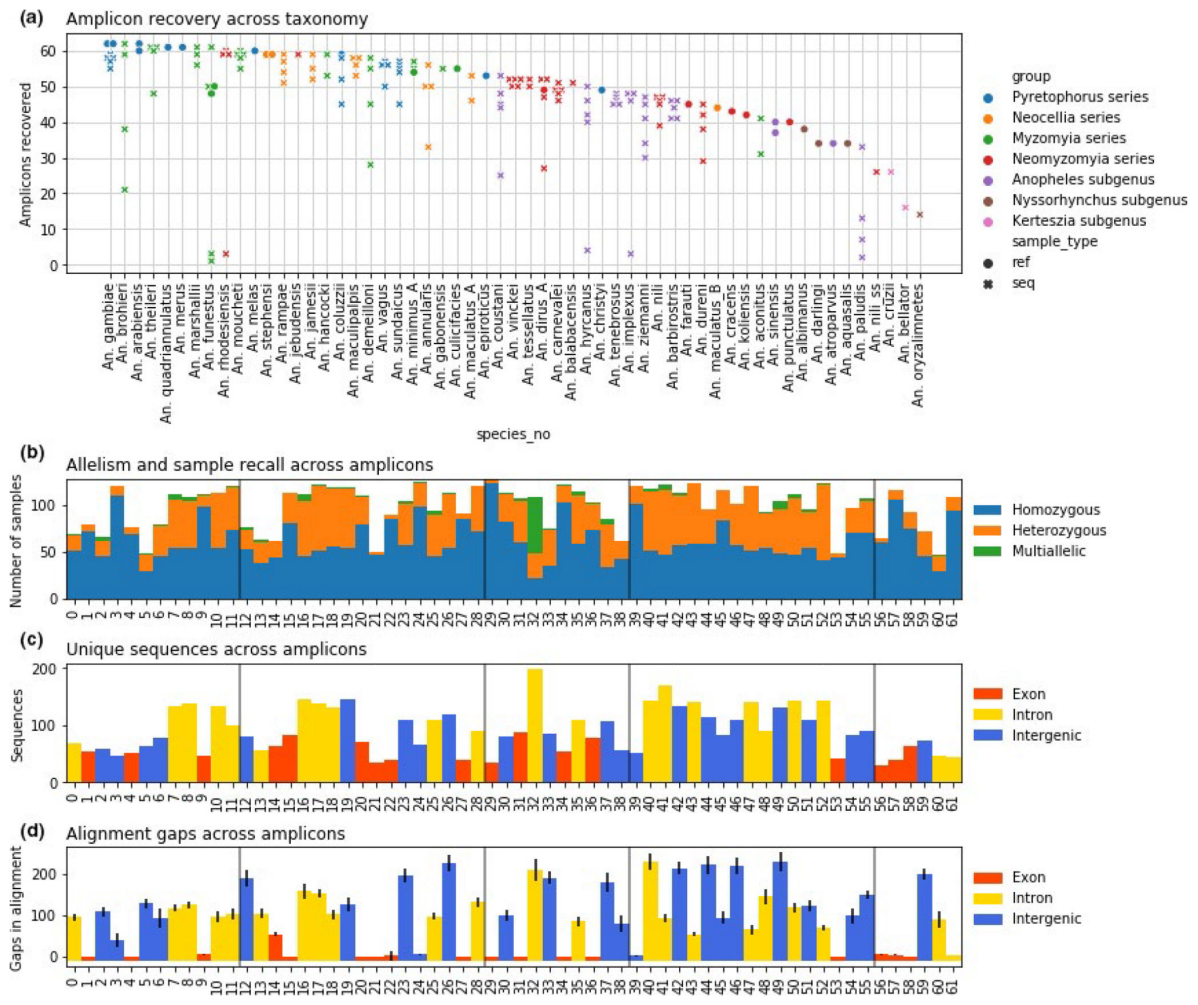
Amplicon sequence recall and variation. (a) Amplicon recovery across *Anopheles* species in 135 sequenced samples and 28 reference genomes, dots correspond to individual samples; colours indicate lineages of *Anopheles* genus. (b) Allele counts per sample for 135 sequenced samples across 62 amplicons. (c) Number of unique sequences in the alignment of sequenced data and reference genomes across 62 amplicons. (d) Number of gaps in the alignment of sequenced data and reference genomes across 62 amplicons; values averaged across aligned unique sequences. Vertical lines in b, d, and d denote chromosome arms (*An*. *gambiae:* 2L, 2R, 3L, 3R, X). Colour in c and d indicates amplicon position relative to AgamP3 genes

Using the panel of 62 targeted mosquito amplicons, we generated sequence data for 135 samples of 40 *Anopheles* species from three continents belonging to three subgenera (*Cellia*, *Anopheles*, and *Kerteszia*) and processed those using the dada2 pipeline. The sequences of an additional 28 samples from 23 species (subgenera *Cellia*, *Anopheles*, and *Nyssorhynchus*) were extracted from published genome assemblies (Supporting Information File S2 tab “sample metadata”), resulting in a total of 161 samples and 57 species in the combined data set. For the 135 samples sequenced using the panel, a total of 6130 loci were recovered (73% of 8370 possible sample-amplicon combinations). At the sample level, this corresponds to 46.1 ± 14.4 (median 50 of 62 possible) amplicons successfully sequenced. The success rate was negatively correlated with the divergence level from *An*. *gambiae*, as this species was used as a baseline for primer design and much variation for *Gambiae* complex was captured in the genome alignment. This trend became noticeable at the subgenus level (Figure 2a), and such amplification failures can be used as phylogenetic signals in the future. For example, sequence data from amplicon number 60 are consistently missing from all *An*. *funestus* samples sequenced so far.

The variation detected in the targeted amplicons was considerable. Out of 6130 loci recovered in 135 sequenced samples, 2104 (34%) were heterozygous and another 175 (3%) were multiallelic. Heterozygosity level (Figure 2b,c) was higher for intergenic and intronic amplicons and lower for exonic amplicons. Most of the amplicons that were recovered from the highest number of sequenced samples (tallest bars in Figure 2b) were also highly variable yielding high proportions of heterozygous calls (orange in Figure 2b) and distinct sequences (bar height in Figure 2c). Thus, we expect that some of the more variable amplicons will be operational in the most divergent lineages, thus resolving species despite a decreased number of successful amplicons. We also examined the number of gaps in the alignment (Figure 2d), as those are indicative of homology loss for more rapidly evolving sequences and thus mark amplicons potentially problematic for conventional phylogenetic analyses. Exonic amplicon sequence alignments tended to be ungapped, while most intronic and intergenic alignments were highly gapped.

### 3.4 Species identification

We anticipate that the panel described here will supplement and perhaps eventually replace morphological and single PCR marker based species diagnostics. Accordingly, we generated amplicon sequence data for about 10% of the described *Anopheles* species to assist with accurate identification of known species, and phylogenetic placement of previously undescribed species. To demonstrate that the panel based assignments surpass what is possible with single marker based assignments, we sequenced two conventional molecular species ID markers, COI and ITS2 (Supporting Information Methods section “COI and ITS2 single marker Sanger sequencing”) using Sanger sequencing for the majority of our samples.

NCBI GenBank and BOLD database searches for COI and ITS2 sequences allowed us to confirm the species identities (or series/subgenus for species that were absent from the databases) and revealed a few mis-labelled samples (Supporting Information File S2 tab “species identification”). Phylogenetic trees reconstructed for COI, ITS2, and amplicon sequencing agreed well on a local level and revealed several groupings of closely related species that were not monophyletic (Supporting Information File S3).

For amplicon panel sequences we developed a species identification method based on sequence clustering with cd-hit-est. The clustering is performed for each amplicon independently, and the results are summarised across all amplicons. The single most important parameter for this analysis is the similarity threshold that defines how divergent sequences in each cluster can be. We tested two approaches for setting the threshold: (i) based on amplicon sequencing data combined with reference genomes (Figure S19) and (ii) using Ag1000g Phase2 haplotypes for *An*. *gambiae* and *An*. *coluzzii*. We tested both sets of thresholds and found the Ag1000g-derived option more informative, as it resolved more species and did not excessively split them into several clusters (Supporting Information Methods section “Distance-based species attribution”, Figure S20).

**Figure 3:**
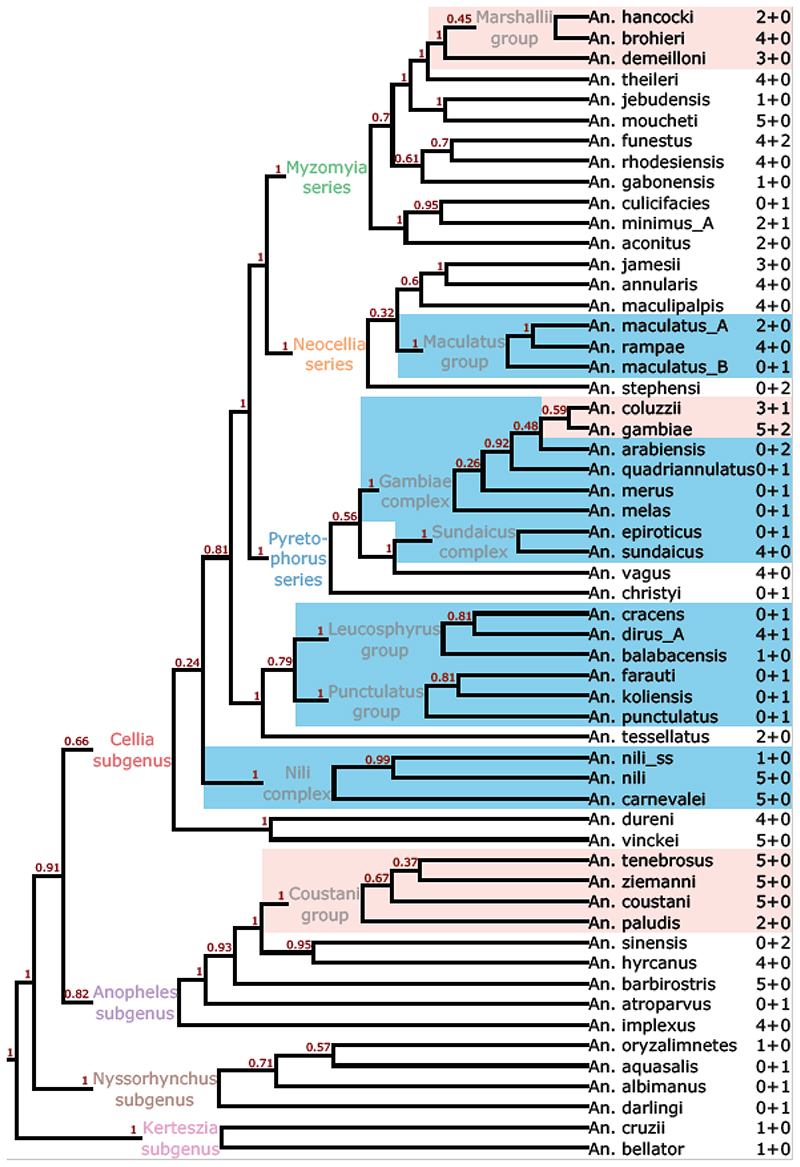
Species identification using the amplicon panel. Species tree cladogram based on 62 amplicons reconstructed in ASTRAL. Support values are given above branches. Groups of closely related species frequently sharing sequence similarity clusters are indicated by colours: blue – unambiguously resolved, red – unresolved. To the right of each species name are the numbers of sequenced samples and reference genomes, respectively, that contributed to this tree (e.g., 2 + 0 indicates two sequenced samples, 0 reference genomes). Series and subgenera are labelled with the same colours as in Figure 2a

Species prediction requires a reference data set with trusted species labels. Here, we created one using a combination of reference genome sequences and quality-filtered amplicon sequence data for wild-caught specimens, retaining 9589 of 10057 (96%) sequences representing 56 of 57 sampled species. The species assignment for each of our samples was based on the most frequently encountered species label across all sequences available for a given sample. This approach resulted in reliable species resolution for most of the species groups and complexes (highlighted in blue in Figure 3). Unsurprisingly, some of the closely related species often co-occurred within clusters (Figure S21), which in some cases resulted in imperfect species resolution (highlighted in red in Figure 3): *An*. *gambiae* and *An*. *coluzzii* (known to have substantial introgression), *An*. *coustani*, *An*. *tenebrosus*, *An*. *ziemanni* (Coustani group), and *An*. *brohieri, An. demeilloni, An. hancocki* (Marshallii group). All of these groups showed evidence of introgression according to COI, ITS2 and amplicon sequencing. Moving forwards, both clustering and phylogenetic trees could be used to identify closest relatives for the new unidentified samples.

To elucidate the power of our panel for resolution of higher level taxonomic relationships, we reconstructed the consensus species tree using ASTRAL based on maximum likelihood trees for individual amplicons for the reference data set (Figure 3). As expected, the overall species tree topology is more similar to that derived from nuclear genomes (Neafsey et al., 2015) compared to the mitochondrial (Hao et al., 2017) and morphological (Harbach & Kitching, 2016) trees. The clade support is higher at both basal and terminal levels with all subgenera, as well as species groups and complexes showing monophyly. Within the *Cellia* subgenus, the analysed series are all monophyletic with the exception of *Neomyzomyia*, which appear as a set of weakly supported sister clades at the base of *Cellia*. *An*. *rhodesensis* and *An*. *jebudensis*, members of the *Neomyzomiya* series, were actually found within *Myzomyia* series according to both amplicon sequencing, COI and ITS2. This also contradicts previous molecular identification results (Ayala et al., 2019), and requires further investigation. It is interesting that the more stringently filtered data set processed with seekdeep yielded a more plausible species tree topology (Figure S18), where the *Neomyzomyia* series was monophyletic.

### 3.5 Population structure

**Figure 4:**
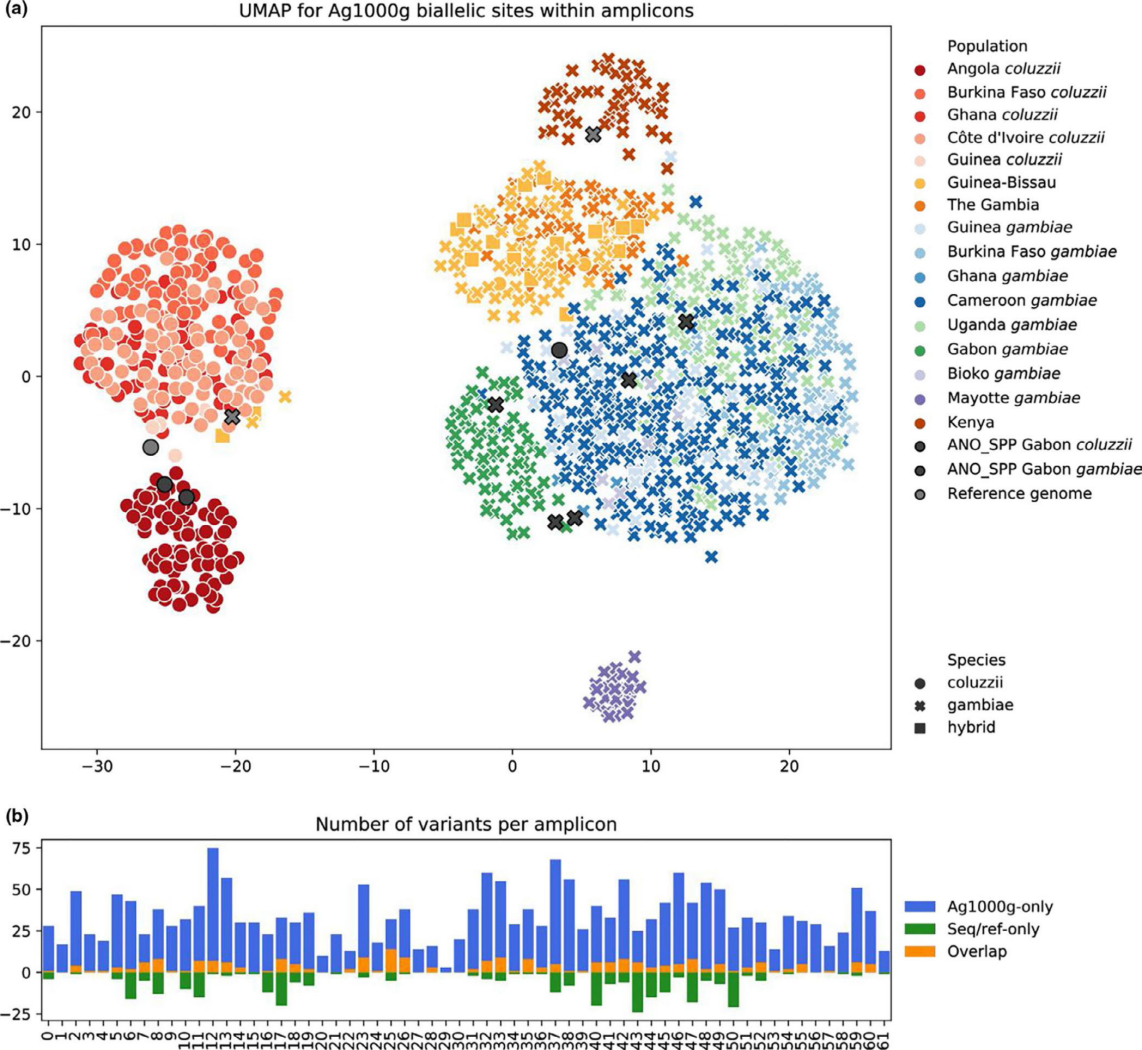
Population structure determined using the amplicon panel. (a) UMAP dimensionality reduction on biallelic sites of Ag1000g Phase 2 data set of 1142 *An*. *gambiae* and *An*. *coluzzii* samples overlapping with positions of 62 amplicons with added amplicon sequencing samples (ANO_SPP) and reference genomes. Colours indicate populations and species, shapes indicate species. (b) Variant counts per amplicon for *An*. *gambiae* and *An*. *coluzzii* samples (seven amplicon sequenced, three reference genomes) compared to Ag1000g Phase 2 biallelic sites (1142 samples)

We next explored the power of the amplicon panel in resolving population structure within species. We generated an in silico targeted amplicon data set based on the whole-genome sequencing data for two closely related and readily crossing species, *An*. *gambiae* and *An*. *coluzzii* (Anopheles gambiae 1000 Genomes Consortium, 2019). Using the Ag1000g Phase 2 variation callset for 1142 individuals we extracted biallelic SNP variants found in each amplicon. A total of 2125 variants were found, and 1417 of those were segregating (i.e., not singletons). Variable sites were distributed across all amplicons and only amplicon 29 did not have any segregating sites among all samples. UMAP dimensionality reduction (Figure 4a) readily split *An*. *gambiae* and *An*. *coluzzii*, and supported the differentiation of the most geographically distant populations (Kenya, Mayotte, Angola). The separation of those three populations was also supported by *F*-statistics (Supporting Information File S4 tab “Fsts”). Diversity estimates (nucleotide diversity, Watterson’s *θ*, Tajima’s *D*, see Supporting Information File S4 tab “diversity”) also correlated well with the values observed from the whole-genome data set. Next, we combined the Ag1000g data with samples from our amplicon sequencing and reference genomes. UMAP allowed for clear species identification and even some level of population attribution (Figure 4a), despite only a fraction of sequence variation captured by amplicon sequencing was used in this analysis (Figure 4b).

To understand the within-species variation patterns outside of the *Gambiae* complex, we assessed the within-species sequence variation detected in amplicon sequencing and in Sanger sequencing of conventional markers (Supporting Information Methods section “COI and ITS2 single marker Sanger sequencing”, Supporting Information File S2 tab “species summaries”). For most species, we had 1–5 individuals that were sequenced using these approaches. On average, amplicon sequencing yielded about 8943 ± 665 bp of sequence data per sample, and revealed 178 ± 111 SNP sites within a species (Figure S17). In comparison, conventional markers yielded far less sequence data per sample: 602 ± 51 bp for COI and 537±143 bp for ITS2, with typically less than 12 variable sites within a species. The exceptions (listed in Supporting Information Methods) were limited to a single marker, that is, COI was diverged while ITS2 was conserved or vice versa, highlighting one problem with single marker approaches: these highly diverse outliers could be interpreted as a single species with one marker, and distinct species if a separate marker was used. Targeting multiple genomic regions helps to address this issue.

Amplicon sequencing informativeness was retained even when fewer amplicons were recovered and the combined amplicon length never dropped below 7kbp for any sequenced species, except *An*. *paludis*, *An*. *nili* s.s., *An*. *cruzii*, *An*. *oryzalimnetes* and *An*. *bellator*—those mostly had <30 amplicons recovered in any sample (Figure 2a) and in some cases, we also failed to generate genome sequence data indicating a likely problem with the source DNA. Moreover, it was possible to discriminate species with higher and lower sampled genetic diversity. We examined a pair of species with lower amplicon recovery. In *An*. *barbirostris* from South-East Asia belonging to Anopheles subgenus, among five sequenced samples 44 ± 3 amplicons recovered, and 21 ± 1 of the recovered amplicons were heterozygous within any given sample. In addition, we found 255 SNPs across 8054 bp all amplicons alignment in this species. In contrast, *An*. *dureni* from Africa, a basal species in *Cellia* subgenus (*Neomyzomyia* series) with four samples sequenced, had only 4 ± 3 heterozygous amplicons in any sample among the 39 ± 7 amplicons recovered, and only 26 SNPs across 7196 bp of all amplicons alignment. In fact, the diversity for *An*. *barbirostris* was comparable to *An*. *gambiae*, where for five sequenced samples 57 ± 2 amplicons were recovered, 27 ± 5 were heterozygous, and 382 SNPs were discovered across 9556 bp all amplicons alignment. Thus, the within-species variation can be recovered persistently across species in both Africa and South-East Asia and for both *Cellia* and *Anopheles* subgenera.

## 4 Discussion

Here, we designed a phylogenetic amplicon panel that simultaneously identifies the species, reveals within species population structure, and detects the presence of *Plasmodium* within sampled mosquitoes for the entire *Anopheles* genus, many of which act as vectors of human malaria. Much of our efforts were focused on automating and reducing the cost of sample processing, such as using a simplified DNA extraction that does not rely on pre-existing kits or product purification, and implementing a multiplex PCR that can be paired with one of the most widespread sequencing technologies, Illumina MiSeq. A number of optimisation and validation experiments also proved that the detection of *Plasmodium* infections within a mosquito was reliable to a certain infection titre (<50 fg/μl or two genomes per aliquot, Figures S7 and S16) despite co-amplification in the same tube with mosquito DNA. Using this approach it is now feasible to process over a thousand individual mosquitoes per MiSeq run, greatly increasing the scale available for vector species surveillance. An additional advantage of this approach is that the mosquito carcasses are preserved after DNA extraction, and can be used for subsequent morphological analyses, for example, when a putative new species involved in malaria transmission (or not) is discovered based on the amplicon sequence results. Without the need for preliminary morphological species identification, our method will allow for high-throughput sample analysis in many different settings of vector surveillance in which a high-quality intact adult specimen may not be available for morphological identification, including larval sampling and CDC light trap collections. We hope this new panel will boost research on vector population structure and dynamics as well as on important vector-related phenotypes such as different host preference or resting behaviour (e.g., Degefa et al., 2017; Dia et al., 2013) across the full range of *Anopheles* species. It will now be possible to forgo time-consuming morphological and/or misleading single marker based assays that are frequently unable to provide species level resolution, as any *Anopheles* mosquito can be sequenced and assessed for its species identity and its *Plasmodium* presence with this panel. Addressing groups of cryptic species and the identification of secondary vectors (Afrane et al., 2016) will be much easier and we foresee that the method could be incorporated into routine vector surveillance in conjunction with other assays assessing parasite drug resistance and blood feed patterns (Daniels et al., 2008; Tedrow et al., 2019). It could also be used for metagenomic assays, studying for example eDNA to assess diversity of species breeding in a water sample.

Mosquito species identification is a key application for the amplicon sequencing panel presented here. To predict species, we performed sequence similarity clustering for each amplicon independently, borrowing from the Barcode Index Number system (Ratnasingham & Hebert, 2013) used by the Barcode of Life project (Ratnasingham & Hebert, 2007), which is focused on COI-based animal species identification. An important choice to be made is the similarity threshold, which depends on maximum expected within-species divergence. Here, we relied on existing continent-scale population sequencing data of Ag1000g, despite some limitations, for example, a lack of indel data. In the future, the values can be adjusted using real sequencing data from more extensive population sampling, preferably from multiple taxonomically distinct species across their ranges. The fact that clustering is performed for each amplicon independently makes it possible to identify species even if some of the targets fail to amplify, which is a substantial improvement compared to single marker approaches, where sporadic PCR or sequencing failures lead to a complete loss of species identity information.

Still, delimitation of closely related species is not possible based on clustering results alone, and calls for additional methods, e.g. dimensionality reduction, such as the UMAP presented in Figure 4a, which enables the differentiation of *An*. *gambiae* and *An*. *coluzzii* despite extremely high allele sharing. In our initial sampling we identified several groups of closely related species (e.g., the Marshalli and Coustani groups, highlighted in red in Figure 3), where larger sample sizes, finer scale analysis, and dimensionality reduction methods are also likely to help disentangle the relationships using this panel. When higher numbers of amplicon sequenced samples become available, missing data (e.g., random amplification failures) will become common, and appropriate methods should be adopted to overcome this. Additional methods could be used for closely related species to discriminate amplicons with significant resolution power from those affected by introgression or incomplete lineage sorting. Obtaining a robust reference data set for species labelling is another important challenge for future research. A solution to that would be accumulation of a curated collection of amplicon sequencing data for mosquito specimens identified using both morphological and established molecular methods. As we generate sequence data for additional species and populations we will also be able to further refine species assignment and geographic origins.

Focusing on both conserved and highly variable regions evenly distributed across the genome allowed us to assign species throughout the *Anopheles* genus, while retaining good resolution for within-species level variation and allowing for independent phylogenetic inference across various chromosome locations. Apart from the basic population structure delimitation demonstrated to be effective here, the data can be used for admixture analyses (O’Neill et al., 2013) or tracing of specific populations through time. The panel can also be used as a triage tool for population genomics in key vector species, allowing rapid scans of large numbers of individuals to identify members of distinct lineages, which can then be whole-genome sequenced (Anopheles gambiae 1000 Genomes Consortium, 2017).

Our study primarily focused on African and South-East Asian representatives of the subgenera *Cellia* and *Anopheles* because they are the most speciose subgenera and the majority of malaria vector species reside in these groups. We also examined limited species from the much less speciose Neotropical subgenera *Nyssorhynchus* and *Kerteszia*. While these demonstrated lower amplicon recovery rate (Figure 2a), this lower recovery appears sufficient to assign species. Over the coming years, we seek to demonstrate the ability of this panel to accurately assign identity for every described mosquito species in the genus and accordingly, we are interested to receive up to 10 confidently identified individual specimens from any described *Anopheles* species to help expand the reference index. When there are extremely large geographic ranges or known population structure, we would also like to include additional specimens representing these variations. Please contact us if you would be interested in assisting in this effort.

At this stage, we have primarily tested the ability of the *Plasmodium* primers to reveal the presence or absence of parasites within the mosquito, but it also reveals the parasite species across the *Plasmodium* phylogeny, as we are able to distinguish between the two species experimentally tested here (*P*. *berghei* and *P*. *falciparum*) by BLASTing the amplicon data against *Plasmodium* spp. mitochondrial genomes. Apart from microscopy and ELISA, a widely used method to detect parasites in mosquitoes is PCR, often using nested primers targeting the18S rRNA gene (Snounou et al., 1993) and alternative approaches aiming to overcome its limitations, for example by targeting higher copy-number mitochondrial COI in a single PCR - achieving detection of 43 fg, or two genomes (Echeverry et al., 2017). The quantification of the infection and identification of *Plasmodium* species are addressed using qPCR, where a multitude of assays have been developed—mostly targeting nuclear 18S rRNA and using various qPCR techniques (Bell & Ranford-Cartwright, 2004; Murillo et al., 2019; Rockett et al., 2011; Rosanas-Urgell et al., 2010). For validation of our amplicon sequencing results, we chose a protocol (Bass et al., 2008; Djouaka et al., 2016) that is widely used in vector surveillance (Ibrahim et al., 2020; Menze et al., 2018; Riveron et al., 2019). Despite the original claim of detection limit of 200 fg or about 10 genomes, our dilution series was effective for less than 50 fg, or two genomes—and we achieved similar sensitivity with amplicon sequencing while also generating all the additional data on the mosquito. Accounting for the lysate dilution and aliquoting we carried out, we estimate that about 1000 parasite mitochondrial genomes (which could possibly represent only a single oocyst) in the context of the whole mosquito DNA extract can be detected. The results of qPCR and amplicon sequencing are well correlated in identifying *Plasmodium* infections in laboratory-infected mosquitoes.

Malaria genomic surveillance is important for both parasites and vectors, with research examining genetic diversity, population structure, and the genetic basis of insecticide resistance in mosquitoes (Donnelly et al., 2016) and drug resistance in *Plasmodium* (Apinjoh et al., 2019). We hope that the addition of this amplicon panel will make it possible to massively increase the scale of much needed characterisation of *Anopheles* species composition, population structure, and infection status as we drive malaria towards elimination.

## Supplementary Material

Supplementary Information

## Data Availability

Raw sequencing data have been made available under ENA study ERP126312, Sanger sequencing data is deposited in NCBI GenBank under accessions MW603491-MW603603 (COI) and MW647367-MW647477 (ITS2). Pipelines and analysis code, together with processed amplicon sequences used for species identification and other data are available on GitHub: https://github.com/malariagen/ano-spp.
