# Supplementary material for: A targeted amplicon sequencing panel to simultaneously identify mosquito species and Plasmodium presence across the entire Anopheles genus
Source: Mol Ecol Resour. Author manuscript; Available in PMC 2022 Jul 2. (PMC7612955; doi:10.1111/1755-0998.13436)
Supplement: Supplementary Information [file EMS146395-supplement-Supplementary_Information.docx]

#

[**Supplementary Methods**](#_wo4ajshjuyno) **1**

[DNA retrieval testing](#_w1y8eqqp70l) 1

[Mosquito primer rebalancing](#_n78io0h4cx3) 10

[Plasmodium primer rebalancing](#_op4qxhk72xd6) 11

[qPCR validation of Plasmodium detection](#_229pyj4avhza) 19

[Molecular Species ID validation using COI and ITS2 single marker Sanger sequencing](#_3gjnh2pnx7cs) 21

[Sequence data processing pipelines design and benchmarking](#_24ujsphmavz0) 24

[Panel applicability on outgroup species](#_325x6gwxyjsg) 26

[Distance-based species attribution](#_7sd7bdugqmic) 27

[**References**](#_7fku823y3h1g) **31**

# Supplementary Methods

## DNA retrieval testing

We have designed a plate-based non-destructive DNA extraction approach for fresh, dried, and ethanol-preserved mosquitoes that minimizes the hands-on time and cost of processing compared to methods and kits that require sample destruction. Besides reduced handling time and costs, another major advantage of our approach is that samples are submerged into a lysis buffer and incubated without shaking or beads, so we are able to preserve key morphological features and if needed can go back to samples of interest post DNA extraction. However, we stress that while morphological features, such as leg and wing banding, are still present post-extraction in their respective tubes or plate wells, body parts often detach from the thorax during sample storage and transport due to static electricity (limbs detach in most cases, abdomens and heads detach occasionally). It is also important to note that this being a proteinase-based approach, internal soft tissues are clarified, leaving only chitinous structures intact. Nevertheless, as our custom lysis buffer is primarily proteinase-based with low concentrations of other detergents, it can be diluted without the need for purification and used as a DNA substrate in all subsequent PCR reactions. In order to identify the best lysis buffer, we tested the performance of several different custom tissue lysis buffers on insectary-reared samples of the *An. coluzzii* Ngousso strain and wild-caught *An. funestus* specimens.

Through a literature search we selected three tissue lysis buffers containing similar reagents (A, D, F), and have modified them into five additional lysis buffers (B, C, E, G). The main modifications were removing salts from buffers with high salt concentrations (B), and adding Tween 20 for its surfactant and membrane disruption properties (C, E, G), as it is less disruptive to enzymes than the commonly used SDS. These lysis buffers were as follows:

- Buffer A [(Santos, Ribeiro, Cabral, & Sperança, 2018)](https://paperpile.com/c/WKXs3W/84jgO): 200 mM Tris pH 8.0, 25 mM EDTA pH 8.0, 0.4 mg/ml proteinase K, 250 mM NaCl, 0.5% SDS
- Buffer B (simplified version of buffer A): 200 mM Tris pH 8.0, 25 mM EDTA pH 8.0, 0.4 mg/ml proteinase K
- Buffer C: buffer B with 0.05% Tween 20
- Buffer D (in-house mosquito lysis buffer used for phenol-chloroform DNA extraction): 10 mM Tris pH 8.0, 100 mM EDTA pH 8.0, 0.2 mg/ml proteinase K
- Buffer E: buffer D with 0.05% Tween 20
- Buffer F (modified from [(Gutaker, Reiter, Furtwängler, Schuenemann, & Burbano, 2017)](https://paperpile.com/c/WKXs3W/d9nSP)): 10 mM Tris pH 8.0, 10 mM EDTA pH 8.0, 0.4 mg/ml proteinase K, 5 mM NaCl
- Buffer G: buffer F with 0.05% Tween 20

We wanted to test the efficiency of DNA retrieval, concentration estimates and PCR efficiency for each buffer. Dried *An. coluzzii* were submerged in 60 µl of each buffer (4 samples per buffer), followed by half of the samples being incubated 2-3h, and the other half being incubated overnight, at room temperature (22-25ºC). As a control, to check if any mosquito (or other) DNA is released from a sample without the use of salts or proteinases, we also incubated 4 samples in 60 µl of ultrapure PCR grade water. After incubation the lysates were transferred to a fresh 96-well plate, and 1 µl of lysate was used to prepare a 1:10 dilution with ultrapure PCR grade water. All subsequent tests were done on this 1:10 dilution plate.

DNA concentration was measured with a Quant-iT™ PicoGreen ® dsDNA Kit, and DNA yields in the full 60 µl lysate were estimated for each buffer and water control for the 2-3h and overnight incubation times in males and females separately (Fig S1). However, due to the high amount of salts, pigmented lysates, and other co-extractions, these are likely to be at best approximate quantifications. In females, which were more similar in size, buffers A, B, C, F and G showed comparable DNA release after overnight incubation (563±132 ng on average), and all buffers showed a lower (12-49%) DNA yield with the shorter incubation time. Unsurprisingly, males showed a lower DNA yield than females in most cases (69-290 ng, 23-66% of their respective female extraction sets after overnight lysis), as males tend to be smaller in size in this *Anopheles* group. A lower yield of DNA was observed in samples submerged in water overnight, probably from DNA solubilizing from damaged tissues and cells, as well as DNA present on the sample’s surface.


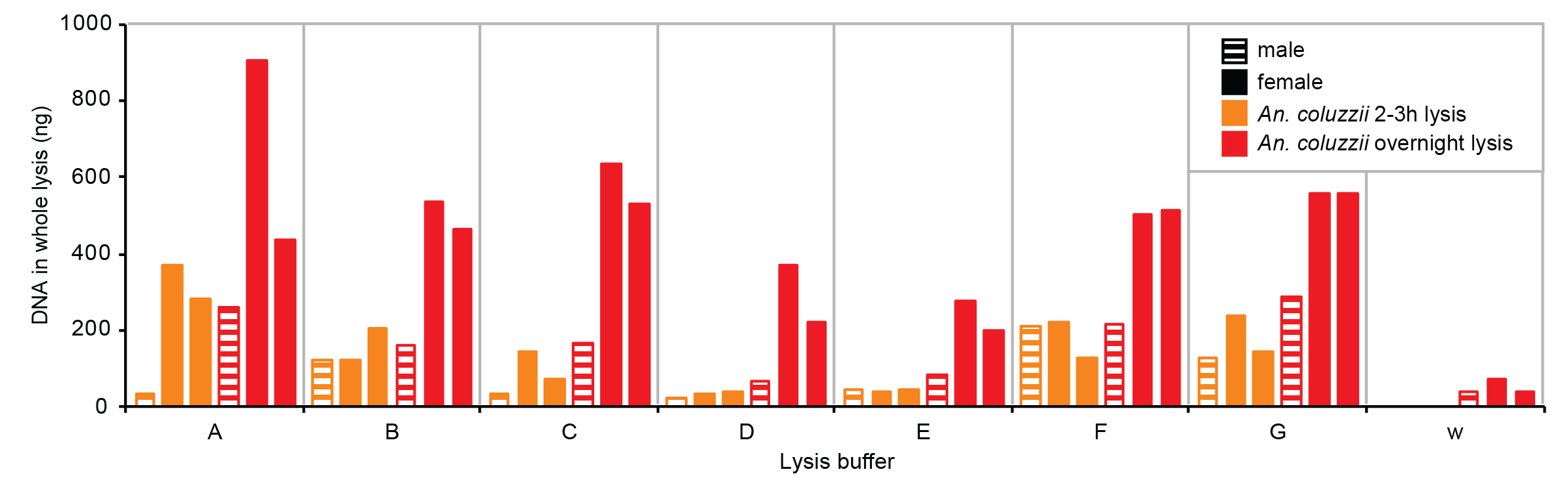


**Figure S1.** DNA yields (ng) released from dry *An. coluzzii* male (horizontal line pattern) and female (no pattern) mosquitoes using various tissue lysis buffers (A-G) and submerged in PCR grade water (w) after a 2-3h lysis (orange) and an overnight lysis (red), measured with a Quant-iT™ PicoGreen™ dsDNA Assay Kit from 1:10 unpurified lysis dilutions.

We also tested these 1:10 lysis buffer dilutions as DNA substrates in a single amplicon PCR reaction. Only one biological replicate per buffer and per incubation time was tested. A species PCR assay able to detect and differentiate species within the *An. gambiae* complex [(Scott, Brogdon, & Collins, 1993)](https://paperpile.com/c/WKXs3W/6GlR) was tested on 16 samples in total. Each 10 µl reaction consisted of 1x GoTaq® Green Master Mix, 1 µM universal forward primer, 1 µM *An. gambiae* specific reverse primer, 1 µM *An. arabiensis* specific reverse primer, and 1 µl of 1:10 diluted lysate as template. PCR cycling conditions were: 95°C for 5 min (enzyme activation, proteinase K inactivation); 30 cycles of 95°C for 30 sec (denaturation) - 50°C for 30 sec (annealing) - 72ºC for 30 sec (extension); 72°C for 5 min (final extension); 10°C hold. After PCR the samples were run on a 2% agarose gel to check for the expected product size (390 bp for *An. gambiae/coluzzii*). For both lysis incubation times successful PCR products were obtained for buffers B, C, F and G, with a very faint band also visible in the 2-3h water incubated sample (Fig S2).


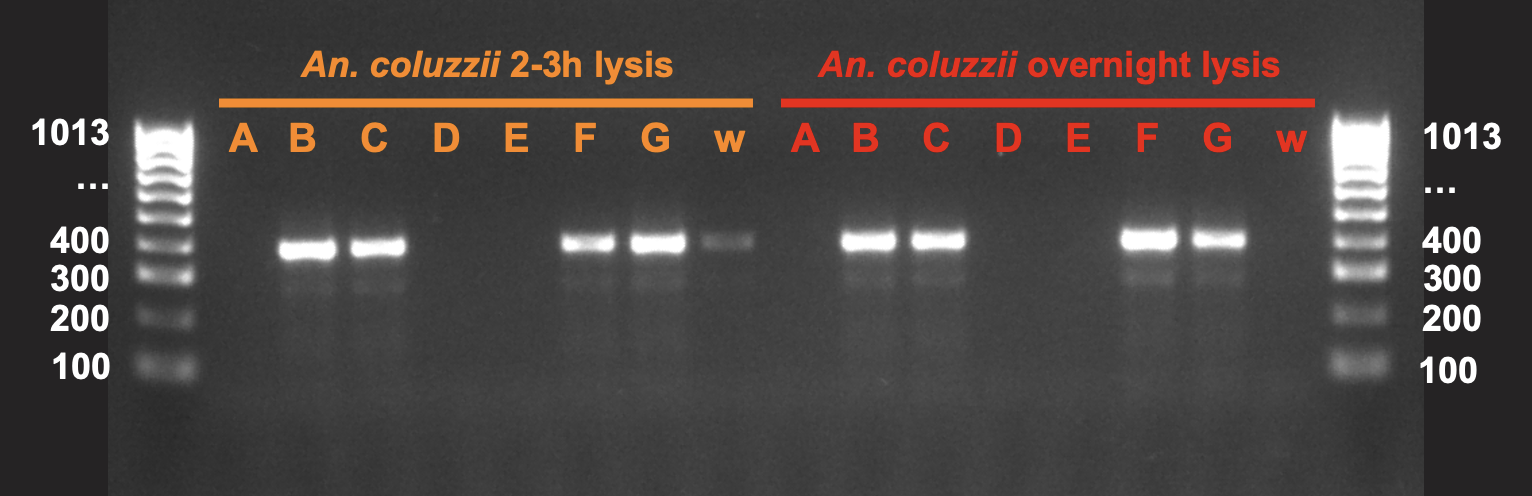


**Figure S2.** Species PCR for diluted unpurified *An. coluzzii* lysates incubated for 2-3h (orange) and overnight (red) in different lysis buffers (A-G) and PCR grade water (w) visualised on 2% agarose gels. Expected product size for *An. coluzzii* is 390 bp. Ladder used was HyperLadder™ 100 bp (Bioline).

From this initial buffer screen we selected three buffers, which we assessed both as unpurified lysate and purified final DNA extracts: buffer A (while it requires a higher dilution than 1:10 in order to consistently work in a PCR reaction without purification, it also has the highest tissue lysis potential and DNA retrieval due to SDS), buffer C and buffer G (both showed similar DNA release as buffer A, efficient PCR on diluted lysis buffer). In spite of good yields and successful PCR amplification, buffers B and F, which contain the same reagent concentrations as buffers C and G respectively, but no detergent (Tween 20), were discarded, because surface tension was high and it was difficult to ensure samples were submerged

Next, we assessed buffers A, C and G for DNA yields, salt co-extraction and species PCR efficiency using both unpurified lysate, as well as purified extracts cleaned using the MinElute 96 UF PCR Purification Kit. Four types of samples were extracted: dried *An. funestus* (8 biological replicates for each buffer, 24 samples total), dried *An. coluzzii* (8 biological replicates for each buffer, 24 samples total), 100% ethanol stored blood-fed *An. coluzzii* (8 biological replicates for each buffer, 24 samples total), and ethanol stored not blood-fed *An. coluzzii* (4 biological replicates for each buffer, 12 samples total). For samples stored in ethanol, ethanol was removed and the samples dried in an oven at 37ºC prior to DNA extraction. DNA extraction was performed as previously described by submerging samples in 60 µl of buffer overnight, however the incubation temperature was raised to 56ºC to increase proteinase K efficiency. After incubation 10 µl was transferred to a new plate, of which 1 µl was added to a plate containing 9 µl of PCR grade water to create a 1:10 dilution plate, and the remaining 50 µl was purified using the MinElute 96 UF PCR Purification Kit following Manufacturer’s protocol with minor modifications that we performed an optional water wash and we eluted in 40 µl ultrapure PCR grade water instead of 20 µl.

DNA concentration in both the 1:10 unpurified lysis dilution and undiluted purified DNA extracts was measured with a Quant-iT™ PicoGreen ® dsDNA Kit, and DNA yields (in ng) for a 50 µl aliquot were estimated and averaged across biological replicates for each buffer and sample type (Fig S3). Based on the PicoGreen Manufacturer’s manual, buffer A contains the most PicoGreen dsDNA signal affecting components, and the concentration of SDS at 10x dilution is still above the recommended maximum acceptable concentration (0.05% instead of 0.01%). Therefore the 1:10 lysis buffer A dilution concentration estimates are likely misleading. Averaged across all samples regardless of species or preservation conditions we see that all buffers perform similarly with a range of 100-300 ng DNA released. We also see that the salts in buffers C and G do not severely affect DNA concentration measurements, as there is no statistically significant difference between the estimated DNA yields from diluted lysate versus purified extracts. However, we still recommend considering these estimates to be only approximate, as other factors could influence the measurements, such as co-extraction of fresh blood meals or eye pigments.


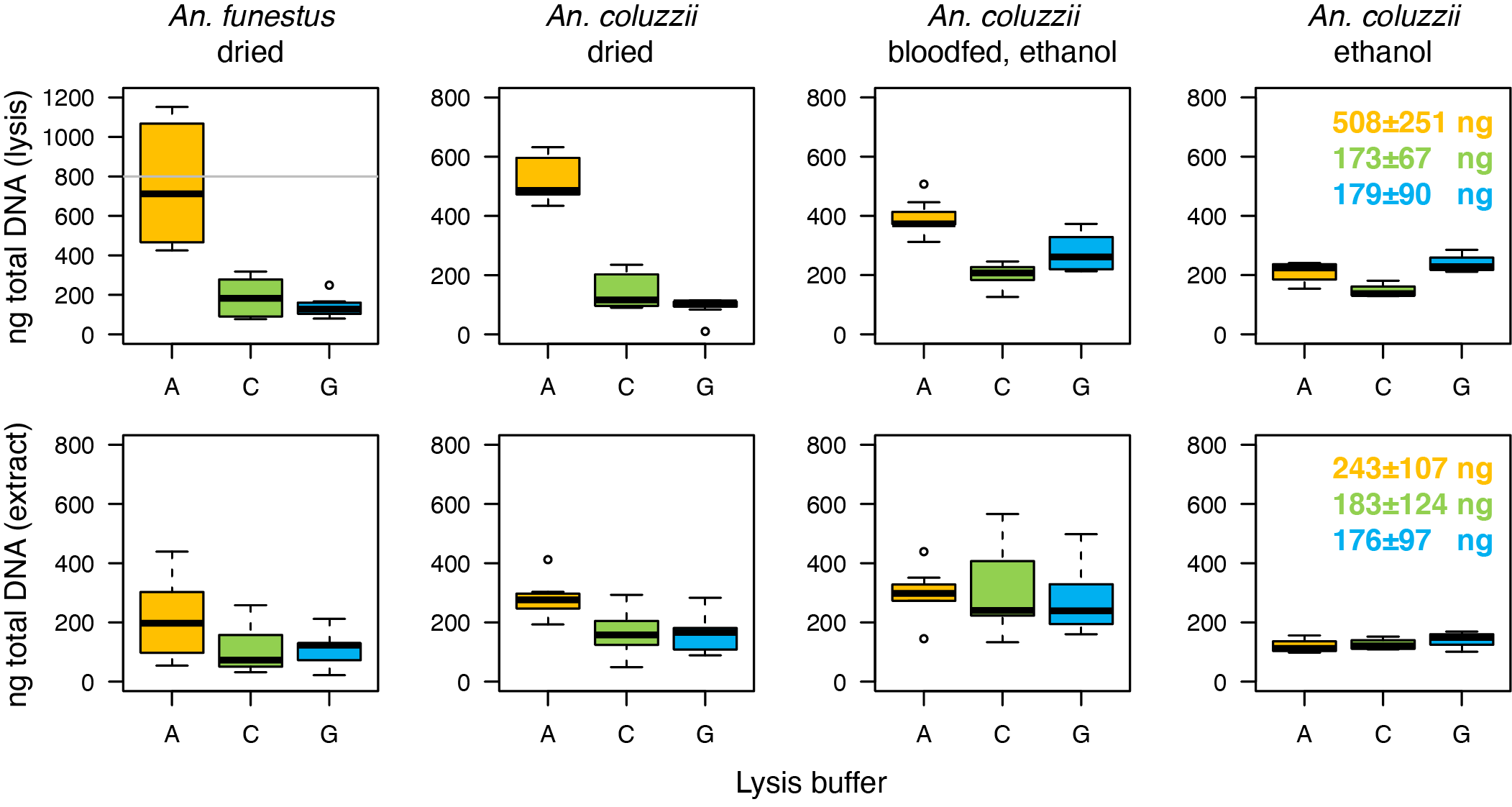


**Figure S3.** Estimated DNA amounts (in ng) retrieved from each sample type using buffers A (yellow), C (green) and G (blue), based on Quant-iT™ PicoGreen™ dsDNA Assay Kit concentration (ng/µl) measurements of 1:10 unpurified lysis dilutions (upper row) and MinElute 96 UF PCR Purification Kit purified extracts (lower row). Each box and whiskers consists of 8 biological replicates, except for *An. coluzzii* in ethanol which consist of 4 biological replicates. The grey line in the first panel (lysis estimation *An. funestus*) denotes the y axis limit of all other panels. Average DNA amounts across all samples extracted with the same lysis buffer (28 for each) and their standard deviations are noted at the end of each row in yellow (A), green (C) and blue (G).

To further evaluate the level of salt and protein co-extractions we measured the MinElute 96 UF purified extracts on a Nanodrop in order to obtain 260/280 (protein) and 260/230 (salt) absorbance ratios (Fig S4). This was used as a proxy for the lysis buffers themselves, as they contain too many Nanodrop-confounding contaminants to be directly measured, and do not contain enough DNA at our 1:10 dilution to be detected on Nanodrop. Based on both absorbance ratios, buffer A underperforms (lower absorbance levels) the other two buffers, followed by buffer G and finally buffer C, for which the range of 260/280 and 260/230 absorbances were closest to theoretical values for clean DNA extracts of 1.8 and 2.0 respectively. This might have to do with the fact that buffer C contains the least number of reagents out of the three buffers.


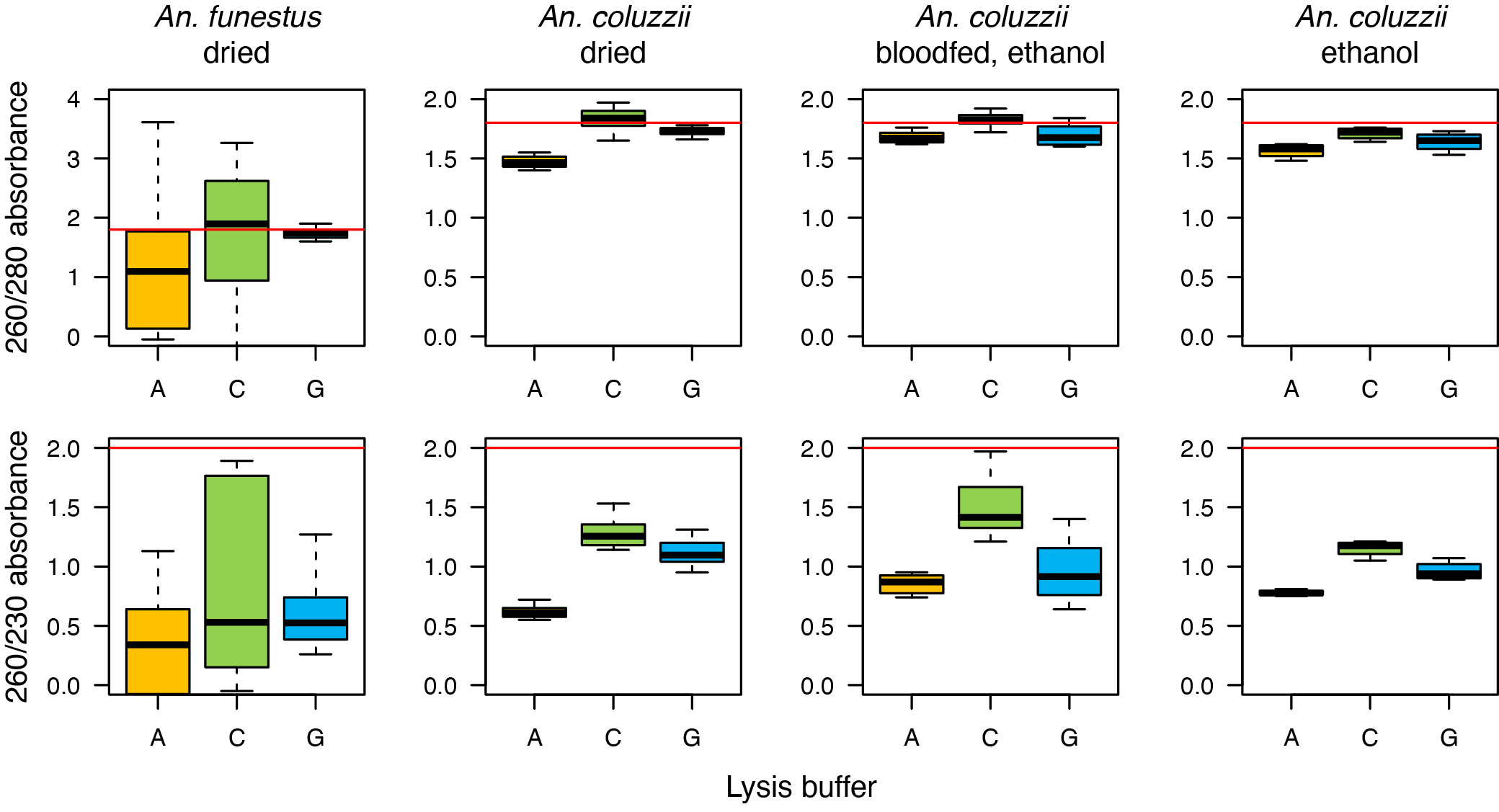


**Figure S4.** Nanodrop 260/280 (upper row) and 260/230 (lower row) absorbance ratios in *Anopheles* samples lysed using buffers A (yellow), C (green) and G (blue) and purified using the MinElute 96 UF PCR Purification Kit. Horizontal red lines denote ideal measurements for pure DNA extracts, while lower values denote co-extraction of proteins (260/280) or organic contaminants and salts (260/230). Each box and whiskers consists of 8 biological replicates, except for *An. coluzzii* in ethanol which consist of 4 biological replicates. For *An. funestus* samples there were 3 samples that showed negative 260/280 ratios, and 3 samples that showed negative 260/230 ratios, as well as one sample that showed an unusually high 260/280 ratio (these samples are not featured due to the y axis cut off).

Finally we wanted to test the efficiency of PCR amplification from these lysis buffers compared to their MinElute 96 UF purified extracts, since doing the PCR on lysis buffer saves £0.48 per sample (cost of the MinElute 96 UF kit per sample/well). We performed a species PCR assay able to detect and differentiate species within the *An. funestus* complex [(Cohuet et al., 2003; Koekemoer, Kamau, Hunt, & Coetzee, 2002)](https://paperpile.com/c/WKXs3W/jmEg+zGTZ), as well as the already mentioned *An. gambiae* complex PCR assay [(Scott et al., 1993)](https://paperpile.com/c/WKXs3W/6GlR). For the *An. funestus* complex PCR assay, each 10 µl reaction consisted of 1x GoTaq® Green Master Mix, 1 µM forward primer, 1 µM reverse primer mix (5 species-specific primers), and 1 µl of target DNA. The DNA template used for both PCR assays was undiluted lysis buffer, the 1:10 lysis buffer dilution, undiluted MinElute 96 UF purified extract, and 1:10 diluted MinElute 96 UF purified extract. *An. funestus* PCR cycling conditions were: 94°C for 2 min (enzyme activation, proteinase K inactivation); 35 cycles of 94°C for 30 sec (denaturation) - 45°C for 30 sec (annealing) - 72ºC for 40 sec (extension); 72°C for 5 min (final extension); 10°C hold. After PCR the samples were run on 2% agarose gels to check for expected product sizes (505 bp for *An. funestus*, 390 bp for *An. gambiae/coluzzii*), the results of which can be found in Fig S5. Unfortunately, during PCR the plate sealing foil in two plates (*An. funestus* dried and *An. coluzzii* dried) buckled up due to heat, causing evaporation below 5 µl in some wells (volume loaded into each well of the agarose gel), and such wells were flagged as failed (red asterisk (*) on Fig S5)). In summary, 1:10 diluted unpurified lysates of buffers C and G performed just as well as 1:10 diluted purified extracts of all three buffers, while occasional failures in purified undiluted extracts suggest an inefficient removal of PCR inhibitors using the MinElute 96 UF plate desalting approach (Table S1).


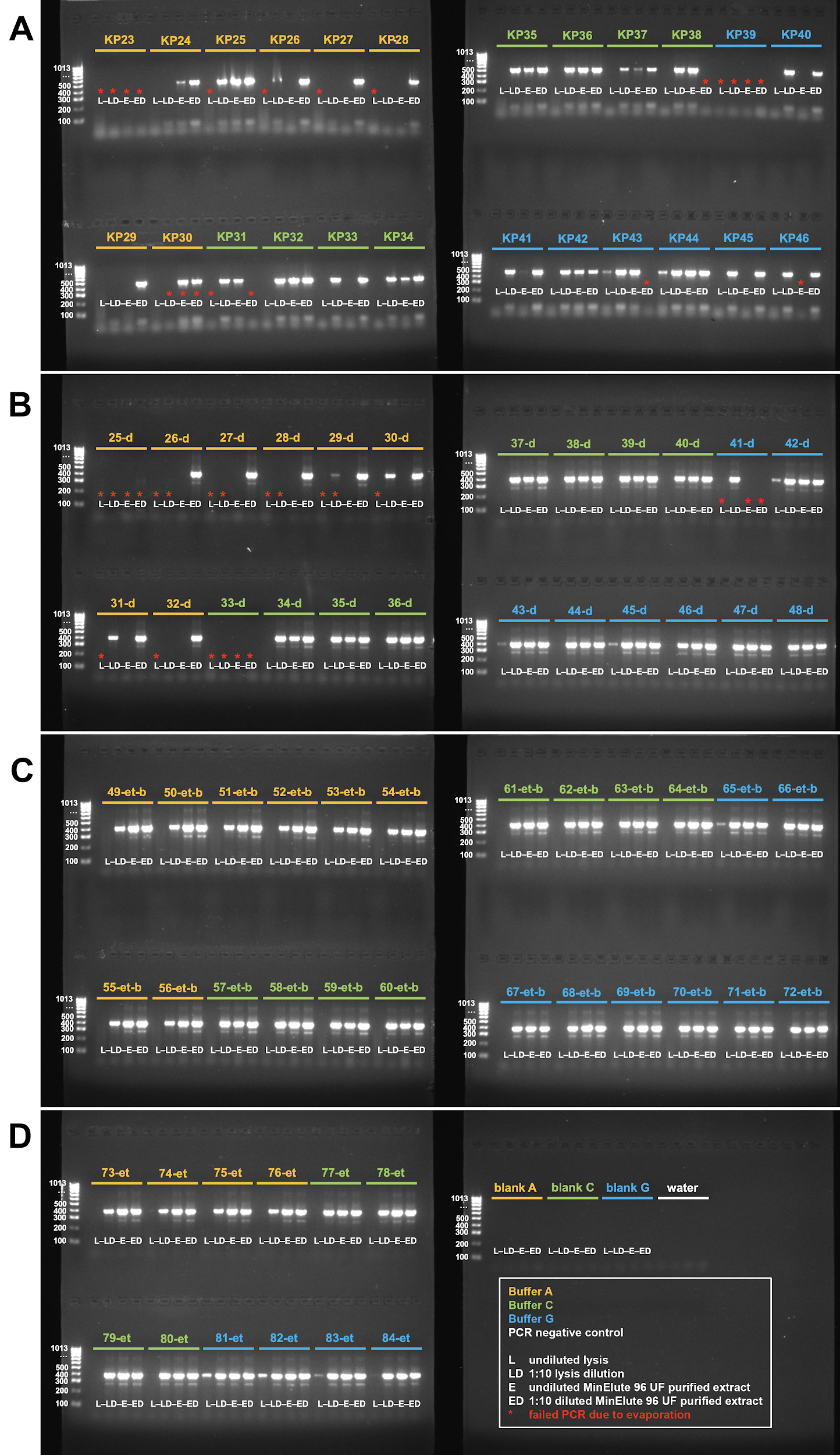


**Figure S5** (previous page). Performance of buffers A, C, and G in PCR. Species PCR for 84 *An. funestus* and *An. coluzzii* samples (laboratory sample IDs above each line) performed using unpurified lysate (L), MinElute 96 UF purified extracts (E), and their 1:10 dilutions (LD, ED) as DNA template, visualised on 2% agarose gels. The panels denote different species and sample preservation conditions prior to DNA extraction: A) wild caught dried *An. funestus* samples, B) insectary *An. coluzzii* dried samples (not blood fed), C) insectary *An. coluzzii* 100% ethanol stored samples (blood fed with fully digested blood meal), D) insectary *An. coluzzii* 100% ethanol stored samples (not blood fed) and lysis buffer and PCR negative controls (blanks). Expected product size for *An. funestus* is 505 bp and for *An. coluzzii* is 390 bp. Ladder used for all was HyperLadder™ 100 bp (Bioline).

**Table S1.** Summary of amplified PCR products versus total successful PCR reactions excluding failures due to evaporation, highlighted in Fig S5 with a red asterisk (*).

| **DNA template** | **Buffer A** | **Buffer C** | **Buffer G** |
| --- | --- | --- | --- |
| Lysis (L) | 0/15 (0%) | 0/26 (0%) | 9/26 (35%) |
| Lysis Dilution (LD) | 16/21 (76%) | 27/27 (100%) | 27/27 (100%) |
| Extract (E) | 14/25 (56%) | 26/27 (96%) | 22/25 (88%) |
| Extract Dilution (ED) | 25/25 (100%) | 25/25 (100%) | 25/25 (100%) |

Taking into account the required number of reagents (Tris, EDTA, Proteinase K and Tween 20) and the results from our quality control measurements (PicoGreen concentration, Nanodrop absorbance, species PCR efficiency), we selected buffer C as our primary DNA extraction buffer. The fully optimized laboratory procedure schematic from mosquito sample plate to Illumina MiSeq sequencing is detailed in Fig S6. The final cost of DNA extraction per sample will depend on the reagent supplier, but we have calculated for our reagents this totals to about £0.09 per sample (reagents only, no plasticware), as detailed in Table S2.


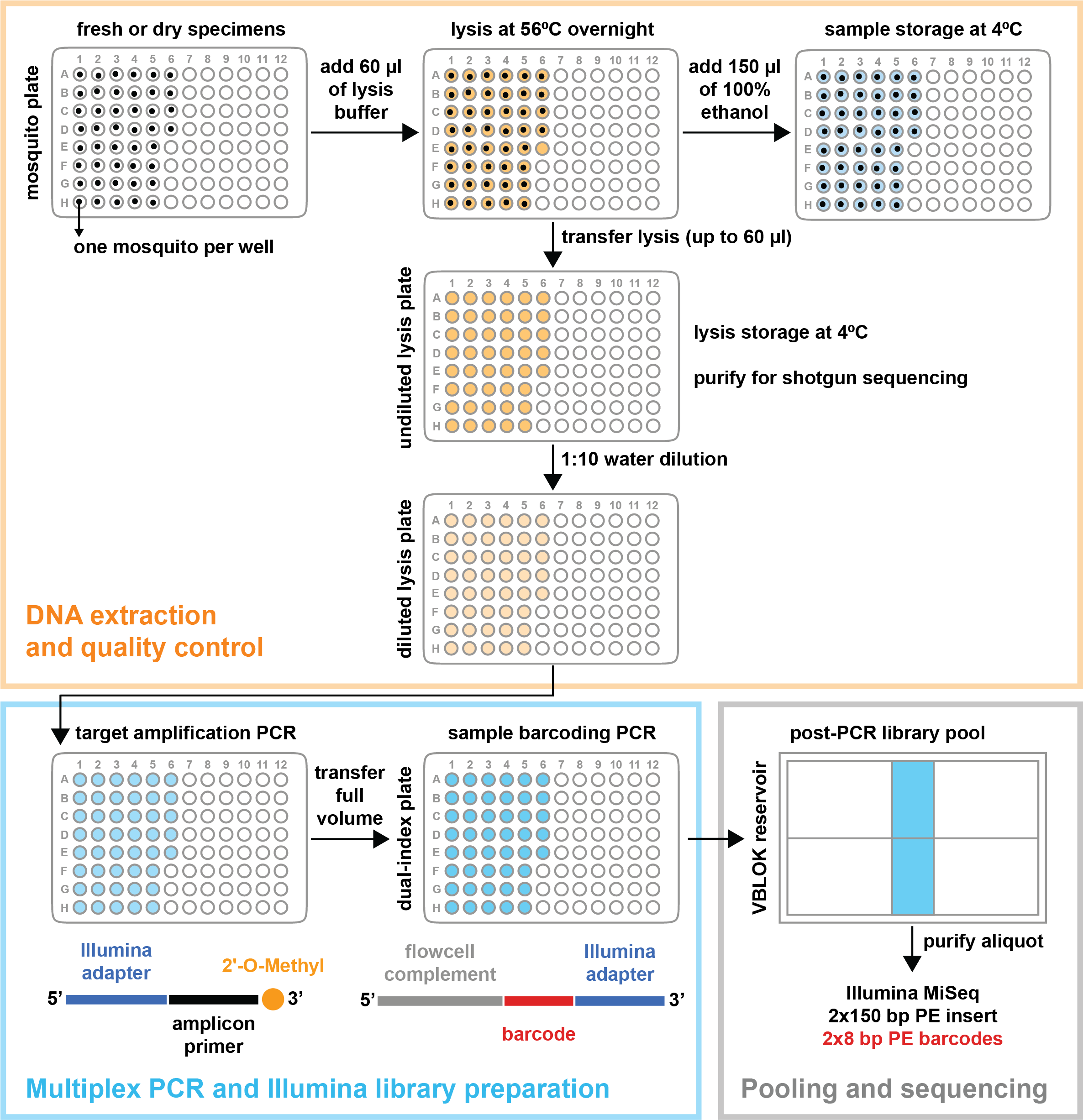


**Figure S6.** Sample preparation and sequencing scheme for the amplicon panel. The different steps of sample preparation are DNA extraction and quality control (orange), multiplex PCR and Illumina library preparation (blue), and pooling and sequencing (grey), with quality control measurements highlighted in shaded boxes.

**Table S2.** Costs of reagents used for preparing and extracting DNA using lysis buffer C. The reported prices are taken from each supplier’s official website as of April 2020, and will vary in different countries and from different providers.

| **Reagent** | **Supplier**  **REF ID** | **Price GBP as of April 2020** | **Total samples per unit** | **Price per sample** |
| --- | --- | --- | --- | --- |
| PCR grade water  (ThermoFisher) | 10977-035 | 15.62 | 9,299 | 0.002 |
| Tris pH 8.0 (1 M) (AppliChem) | A4577,0500 | 22.83 | 41,667 | 0.001 |
| EDTA pH 8.0 (0.5 M) (AppliChem) | A4892,0100 | 18.71 | 33,333 | 0.001 |
| Tween 20 100% (Sigma-Aldrich) | T2700-100ML | 27.70 | 3,333,333 | 0.000 |
| Proteinase K 20 mg/mL (Invitrogen) | 10665795 | 65.75 | 1,042 | 0.063 |
| Ethanol 100% (Sigma-Aldrich) | 51976-500ML-F | 78.30 | 3,333 | 0.023 |

## Mosquito primer rebalancing

In Target Amplification PCR, the use of equimolar concentrations of primers for each target within a panel tends to result in a significant imbalance in the numbers of reads obtained from each of the targets. There is no obvious mechanism to predict primer pair performance *a priori*, so in order to normalise the numbers of reads obtained from each primer pair we must first determine the read depths obtained from an equimolar primer pool and use these data to drive an optimisation process where we increase the concentration of the underperforming targets and decrease the concentration of the overperforming ones (we call this a *rebalanced* primer pool). Note that the priming efficiency of a given primer pair will tend to be determined by the weaker of the pair so we do not need to allow for differences within primer pairs as well (this would significantly increase the complexity of the rebalancing process). We use an empirically-validated rule-of-thumb that borrows heavily from the method described in [(Nguyen-Dumont et al., 2013)](https://paperpile.com/c/WKXs3W/8EX7y). Specifically:

1. The *read fraction* (reads obtained for a particular target ÷ total number of reads) is obtained for each primer pair in the equimolar pool for each individual sample within a set of representative samples, and the median of these read fractions is determined.
2. These *median read fractions* are then divided by the sum across the pool so that they sum to unity.
3. The median read fractions are transformed into *pooling weights* (i.e. the relative amount of the primer pair that needs to be added to the pool in order to compensate for its relative under-/overperformance) as *median read fraction^-0.561^*.
4. In order to pool primers, the pooling weights need to correspond to a range of volumes of the individual stock primer pairs that can be accurately pipetted. We generally scale the pooling weights so that the minimum weight is 1 by dividing each weight by the minimum weight within the pool.
5. Finally, we ‘clip’ the pool weights to a maximum of 10 as this rule-of-thumb will tend to produce very high weights for particularly weak primer pairs, which will tend to dominate all the other primer pairs if used naively. This has the unfortunate consequence of reducing yields from weak primers, but it is necessary.

## *Plasmodium* primer rebalancing

For the panel described in this paper, there was an additional complication in that the *Plasmodium* targets (P1 and P2) are present at a copy number that is orders-of-magnitude lower than the *Anopheles* targets. Compensating for this phenomenon required empirical titration of *Plasmodium* primers against the rebalanced *Anopheles* primer pool in order to determine the amount of additional primer required to overcome this. The testing was done with serial dilutions of *Plasmodium*-mosquito DNA mixtures and lab-infected mosquitoes sampled at various time points post feed.

Serial dilutions were used combined with a broad range of primer concentrations. In the first experiment, we tested serial dilutions of *P. falciparum* (15 pg/µl - 1.5 fg/µl in decrements of 10x) with *An. gambiae* (1.5 ng/µl) DNA - ratios of 1:100 to 1:1,000,000. Primer concentrations were set to 1x, 3x, and 9x compared to initial mosquito primer concentration of 300 pM for both P1 and P2 in all possible combinations. The results of this experiment (Fig S7, top) showed that P2 was much more efficient than P1 and that sensitivity was insufficient for both pairs of primers.


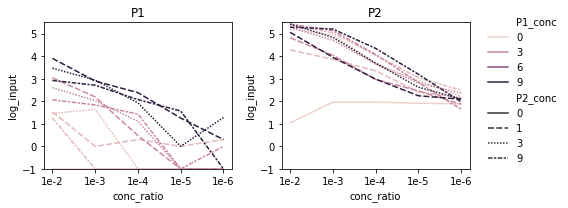


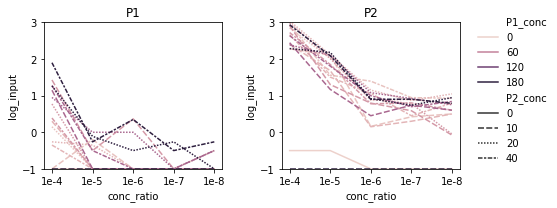


**Figure S7.** Plasmodium primers sensitivity. Log scale read counts for serial dilution of *P. falciparum* DNA. Primer concentrations are given compared to 300 pM. Top: experiment 1. Dilution range: 15 pg/µl (1e-2 parasite to mosquito ratio) - 1.5 fg/µl (1e-6 ratio). Primer concentrations 0x, 1x, 3x, 9x for both P1 and P2. Bottom: experiment 2. Dilution range: 50 fg/µl (1e-4 parasite to mosquito ratio) - 5 ag/µl (1e-8 ratio). Primer concentrations (compared to 300 pM): P1 - 10x, 20x, 40x, 80x, 160x, P2 - 10x, 20x, 40x.

For the second experiment, we aimed to test higher primer concentrations combined with lower *Plasmodium* concentrations to determine the parasite detection limits of the amplicon sequencing approach. The following primer concentrations were tested: 10x, 20x, 40x, 80x, 160x P1 and 10x (pools 1-5), 20x (pools 6-10), 40x (pools 11-15) P2. Serial dilutions of *P. falciparum* (50 fg/µl - 5 ag/µl in steps of 10x) with *An. stephens*i (0.747 ng/µl) or *An. coluzzii* (1.238 ng/µl) DNA were made - approximate ratios of 1:10,000 to 1:100,000,000. Limit of parasite detection was reached at 5 fg/µl for P1 and at 0.5 fg/µl for P2 (Fig S7, bottom). P2 was again much more efficient than P1, even in case of 16x excess of P1 (pool 5).

Using the same 15 combinations of primer concentrations (10x-160x P1 and 10-40x P2), we assessed *Plasmodium* detection in 40 lab-infected mosquitoes: *An. stephensi* were fed with a *P. falciparum* gametocyte culture and sampled at 0 hours, 24 hours, 3 days and 9 days after feed. Unfed mosquitoes were used as control and 8 specimens were included for each time point, including controls. Infection levels were as expected with every fed sample showing high read counts at days 0-1 post infection due to the parasites present in the bloodmeal followed by lower read counts and some negative samples at days 3 and 9 as no more bloodmeal parasites are present and any detection reflects oocysts and/or sporozoites. In uninfected samples, parasite detection was extremely low, but for a few samples at particular primer concentrations P2 did exceed the 10 parasite reads that we consider background level detection (Fig S8). As before, P2 was much more efficient than P1, with 1:10 P1:P2 ratio reached for 160x P1 and 10x P2. Unfortunately, a combination of 160x P1 and 10x P2 failed, presumably due to amplification inhibition at high primer concentrations (Fig S9).


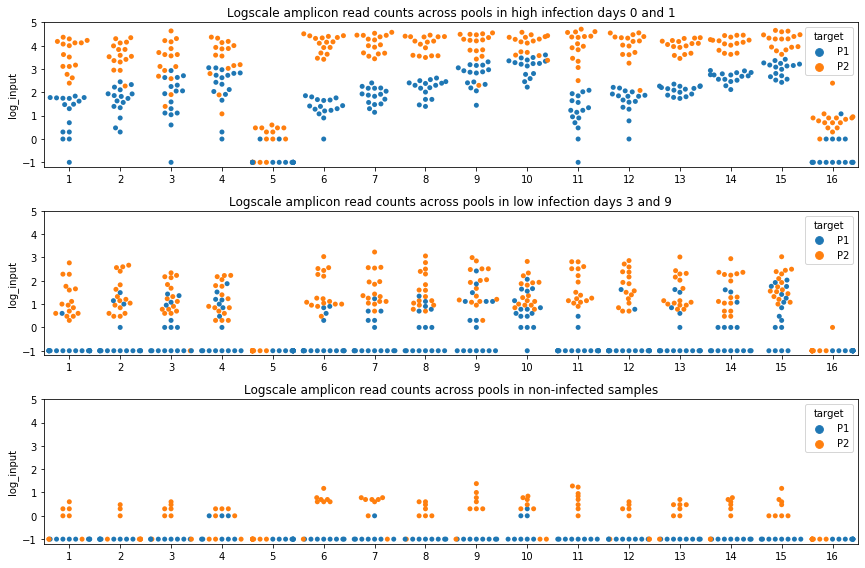


**Figure S8.** Primer concentration effects on various infection levels. Log scale read counts for *Plasmodium* primers P1 and P2 across varying primer concentrations (compared to 300 pM): P1 - 10x, 20x, 40x, 80x, 160x, P2 - 10x (pools 1-5), 20x (pools 6-10), 40x (pools 11-15). Subplots: high infection, low infection, uninfected samples.


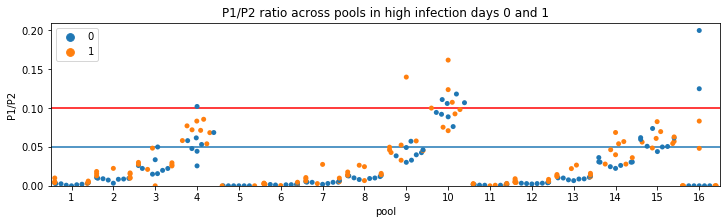


**Figure S9.** Plasmodium primer balance across varying primer concentrations (compared to 300 pM): P1 - 10x, 20x, 40x, 80x, 160x, P2 - 10x (pools 1-5), 20x (pools 6-10), 40x (pools 11-15).

For further optimisation experiments, another set of lab-infected mosquitoes was used in which experiment-level information on infection intensity and prevalence was known based on microscopy. As before, *An. stephensi* were fed with a *P. falciparum* gametocyte culture, and two feed experiments were included - feed15, which had a prevalence of 60% and an average oocyst intensity of 5 per gut; and feed16, which had a prevalence of 95% and an average oocyst intensity of 18 per gut. As before, 8 control uninfected mosquitoes were included. Feed 15 was sampled at days 0, 8, and 13/14 and Feed 16 was sampled at day 0 and 9. Each time point included 16 individual mosquitoes for a total of 88 samples.

In an attempt to improve primer balance, we fixed the concentration of the less efficient primer P1 to 80x (as higher concentrations presumably inhibited amplification in the previous experiment) and we tested 10x and 5x concentrations of the more efficient primer P2. As a control, we prepared an amplicon panel consisting of only primers P1 and P2 at 10 nM concentration (i.e., ~33x). P2 was always more efficient than P1, as observed from P2/P1 read count ratios: about 20 times for 10x P2 (panel 8010), and about 4 times for 5x P2 (panel 805), while the P1-P2 only panel (plas) had a ratio close to 1:1 (Fig S10 left). Interestingly, in the repeated attempt of the same primer conditions applied to the same samples, the P2/P1 read count ratios shifted twofold: ratio of 10 for 10x P2, ratio of 2.5 for 5x P2 (Fig S10 right). For both primer balances, read counts are as expected: high at day 0, lower at later days. At lowest infection point, infection levels were estimated as 78% (panel 8010) or 69% (panel 805) for feed15 (95% rate by dissection) and 56% (panel 8010) or 50% (panel 805) for feed16 (60% rate by dissection). Background read counts in uninfected samples were low, and generally did not exceed 10 reads - a threshold set in the DADA2 pipeline (Fig S11). Further choice between these two concentrations was informed by qPCR results outlined in the next section.


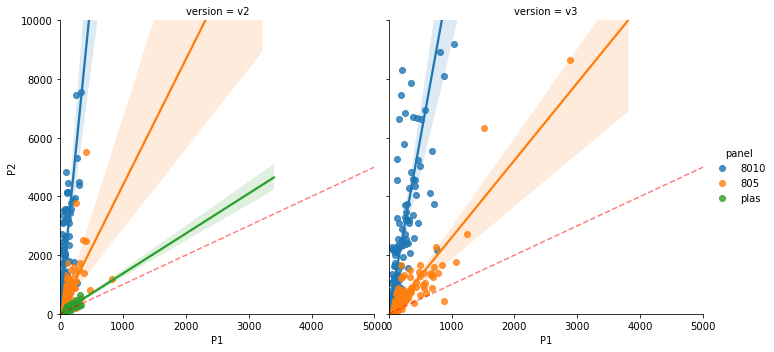


**Figure S10.** Effect of *Plasmodium* primer concentrations on amplification balance. Left: experiment 1, primer concentrations: 8010 - 80x P1 & 10x P2 (ratio 21.5, R=0.76); 805 - 80x P1 & 5x P2 (ratio 4.3, R=0.70), plas - P1 and P2 only (ratio 1.4, R=0.93), ano - mosquito primers only (ratio -0.05, R=0.05). Right: experiment 2, primer concentrations: 8010 - 80x P1 & 10x P2 (ratio 10.9, R=0.95); 805 - 80x P1 & 5x P2 (ratio 2.5, R=0.95). Dotted red line - ratio = 1.


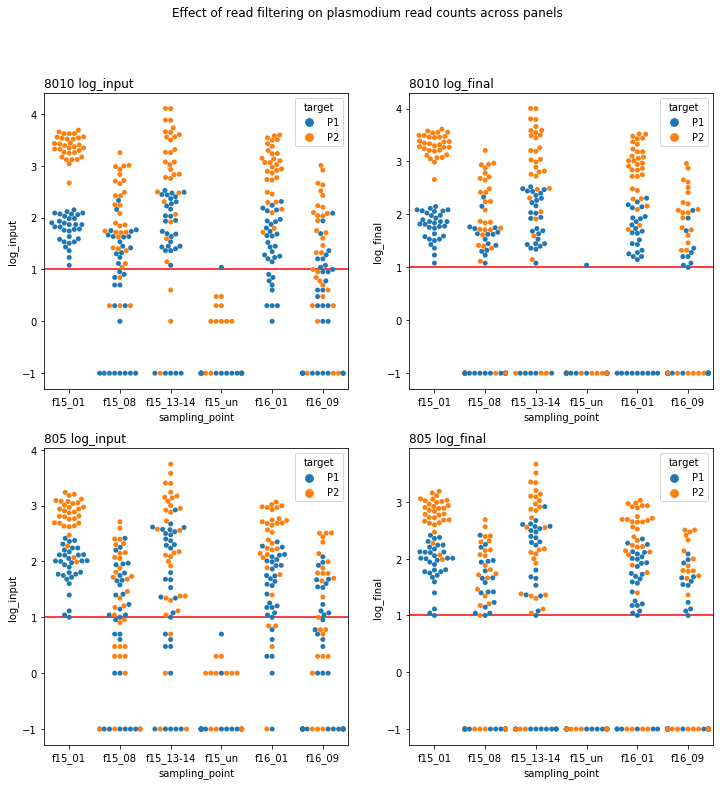


**Figure S11.** *Plasmodium* infection detection with amplicon sequencing. Total read counts (left) compared to post-filtering read counts (right). Colours denote primers. Primer concentrations: 8010 - 80x P1 & 10x P2 (top); 805 - 80x P1 & 5x P2 (bottom). Red line: pipeline filtering cutoff of 10 reads. Within panels, sampling points are split by feed (f15 or f16) and day (1, 8, 9, 13/14, or un for uninfected) - 16 samples per point, except 8 uninfected samples. Highest prevalence and intensity expected when bloodmeal is still present (day 1), followed by decrease in prevalence and intensity during early oocyst formation (days 8-9) and increase in intensity during late oocyst/sporozoite (days 13-14).

We also used the same lab-infected mosquito samples to test several PCR cycling conditions and assess if *Plasmodium* detection could be improved without impairing mosquito primer performance. We tested Sample Barcoding PCR “subcycling”, which differs from standard Sample Barcoding PCR cycling by the use of an oscillating temperature in the annealing/extension phase of the PCR, thus hoping to improve the primer balance [(Liu & Sommer, 1998)](https://paperpile.com/c/WKXs3W/MkOt). This is proposed to help when amplifying multiple targets with widely varying %GC contents as the lower extension temperatures required for lower %GC targets are suboptimal for higher %GC targets and vice versa, so the oscillating temperature enables a compromise to be achieved between the requirements of the different targets. Subcycling PCR was carried out as follows: 95°C hold (PCR plate transferred directly from 4°C cooled Mosquito deck onto thermocycler block, then rest of protocol commenced); 31 cycles of 95°C for 20 seconds (denaturation) followed by annealing/extension using 4 cycles of 68°C for 15 seconds & 60°C for 15 seconds per cycle (i.e. a 4 cycle oscillation is nested within each of the 31 PCR cycles); 68°C for 3 minutes (final extension) followed by a 4°C hold. As a result, the efficiency of P1 increased, while the averaged mosquito yield decreased significantly - especially for the samples with lower read counts under standard conditions (Fig S12, top). Thus, standard cycling was retained. We also tried to decrease the annealing/extension temperature in Target Amplification PCR from 55°C to 51°C. The efficiency of P1 increased, but unfortunately the mosquito amplicon yields decreased significantly (Fig S12, bottom). The extent and direction of changes in read counts differed between mosquito primers as well, suggesting heterogeneity in optimal annealing temperatures.


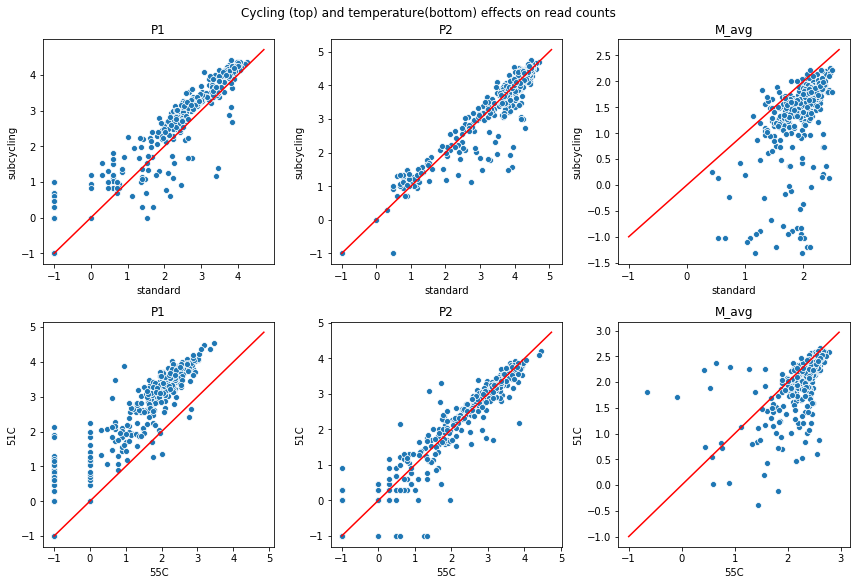


**Figure S12.** Sample Barcoding PCR standard cycling and subcycling (top) and Target Amplification PCR annealing/extension temperature (bottom) effect on log scale read counts. P1, P2 - individual *Plasmodium* primers, M_avg - read count averaged across 62 mosquito targets. Primer concentrations 80x P1, 10x P2.

We also compared our non-destructive DNA extraction approach to a full lysis in buffer C with tissue grinding to test if *Plasmodium* DNA is more efficiently released with mosquito tissue destruction. After initial non-destructive DNA extraction with buffer C (extraction 1), the specimen was ground by adding metal beads to each well and vortexing, followed by a re-extraction using a fresh 60 µl aliquot of buffer C (extraction 2). PicoGreen estimates of total DNA yields show that 52 to 73% of the total DNA was retrieved in extraction 1, suggesting on average we retrieve 64% of the total DNA using our non-destructive incubation approach. Read counts suggest that extraction 1 yielded more DNA (both mosquito and parasite) than subsequent extraction 2, and primer balance was not affected by the extraction (Fig S13). Thus, another evidence of sufficient efficiency of the non-destructive extraction was obtained.


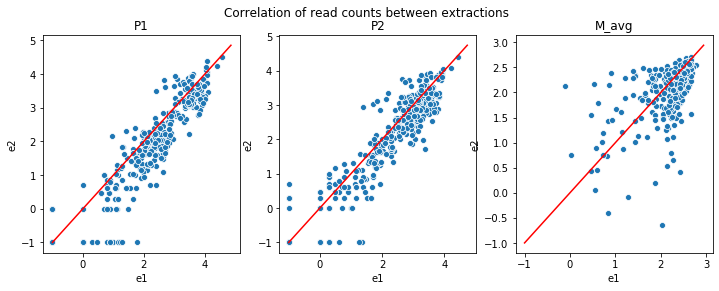


**Figure S13.** Non-destructive (e1) and subsequent destructive (e2) DNA extraction effects on log scale read counts. P1, P2 - individual *Plasmodium* primers, M_avg - read count averaged across 62 mosquito targets. Primer concentrations 80x P1, 10x P2.

While testing various conditions on a single sample set, multiple replicates were generated. Good correlation of read counts for the same samples between conditions suggest the robustness of parasite detection, especially for high levels of infection (Fig S14).


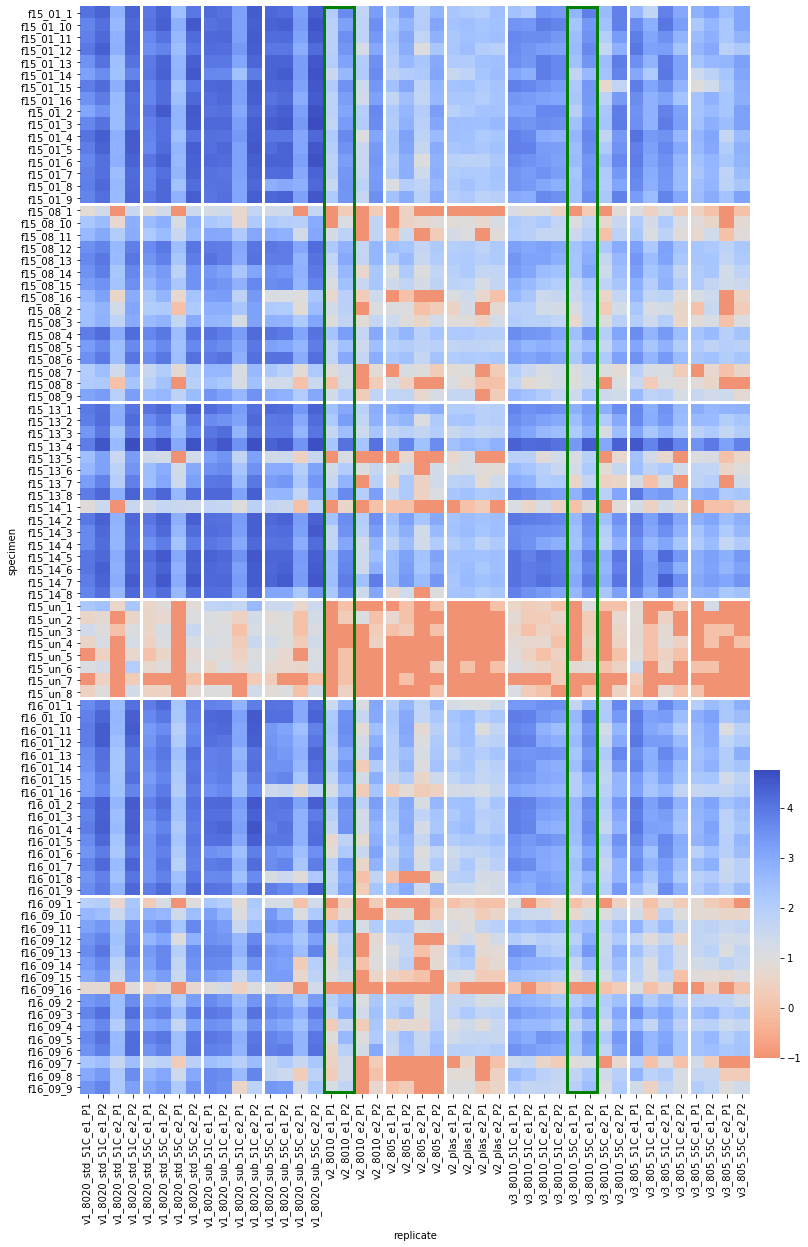
**Figure S14** (previous page). Log scale read counts for various conditions (columns) tested in 88 mosquito samples (rows). Final set of conditions highlighted in green. Sample names consist of feed ID, day post infection (un for uninfected) and internal mosquito number. Replicate names summarise primer concentrations, other conditions used, and finally primer names.

## qPCR validation of *Plasmodium* detection

In order to validate the results of *Plasmodium* detection in the lab-infected *An. stephensi* samples, we performed a well established *Plasmodium* qPCR assay (adapted from [(Bass et al., 2008)](https://paperpile.com/c/WKXs3W/sJT4) with modifications from [(Djouaka et al., 2016)](https://paperpile.com/c/WKXs3W/ovCM)), selected for its extensive use in testing for *Plasmodium falciparum, vivax, ovale* and *malariae* presence in wild caught mosquitoes ([(Ibrahim et al., 2020; Menze et al., 2018; Riveron et al., 2019)](https://paperpile.com/c/WKXs3W/LEt15+Dfl1I+W13rM) to note a few recent publications that utilize this assay). This TaqMan qPCR assay amplifies a 74 bp fragment of the 18S rRNA gene in all four human *Plasmodium* species and uses two probes that allow discrimination of *P. falciparum* (6FAM) from *P. vivax*, *P. ovale*, and *P. malariae* (VIC). Each 10 µl qPCR reaction contains 1 µl of DNA substrate (pure *Plasmodium* DNA or 1:10 mosquito lysis dilution), 1x SensiMix II Probe, 800 nM of each primer (PlasF - GCTTAGTTACGATTAATAGGAGTAGCTTG, PlasR - GAAAATCTAAGAATTTCACCTCTGACA), and 200 nM of each TaqMan MGBNFQ Probe (Falcip - TCTGAATACGAATGTC - 6FAM dye at 5' end, OVM - CTGAATACAAATGCC - VIC dye at 5' end). Amplification was run on a LightCycler® 480 Instrument II (Roche) with the following cycling conditions: 95°C for 10 min (initial denaturation); 40 cycles of 92°C for 15 sec (denaturation) and 60°C for 1 min (annealing and extension with fluorescence capture); final cooling to 10°C.

We applied this assay to DNA extractions from lab infected mosquitoes described in the previous section and to a serial dilution of *P. falciparum* DNA (5 pg/µl, 500 fg/µl, 50 fg/µl, 5 fg/µl), and compared the observed 6FAM (*P. falciparum* specific) fluorescence levels to log scale read counts from selected amplicon sequencing replicates (Fig S15, Fig S16). Despite the fact that sensitivity limit for the assay was established as 200 fg (10 genomes) in the original publication [(Bass et al., 2008)](https://paperpile.com/c/WKXs3W/sJT4), we observed significant differences in fluorescence signal between 50 fg and 5 fg samples (Fig S16). Given that we extract DNA in 60 ul volume and then perform 1:10 dilution, a single parasite infecting a single mosquito would yield 25 fg/600 ul = 40 ag/ul concentration, which is not detectable with qPCR. At 1,000 parasites per mosquito, which roughly equates to a single mature oocyst, we reach a level of 40 fg/µl, or 1-2 parasite genomes/µl, which is detectable with qPCR. In amplicon sequencing, P1 was less efficient, so its concentration was set to 80x (higher concentrations occasionally resulted in amplification inhibition). Still, its sensitivity was not sufficient to identify some of the lower infection samples detected by qPCR at days 3-9. In contrast, P2 was highly efficient and we tested three concentrations for it. At 20x P2, a slightly elevated number of false positives was observed (amplicon sequencing reads present in absence of qPCR signal) (Fig S15). At 5x P2, false negative rate was increased manifesting as low amplicon sequencing read counts in presence of qPCR signal (Fig S16). In both experiments, 10x P2 demonstrated the optimal balance with higher level of concordance between amplicon sequencing and qPCR.


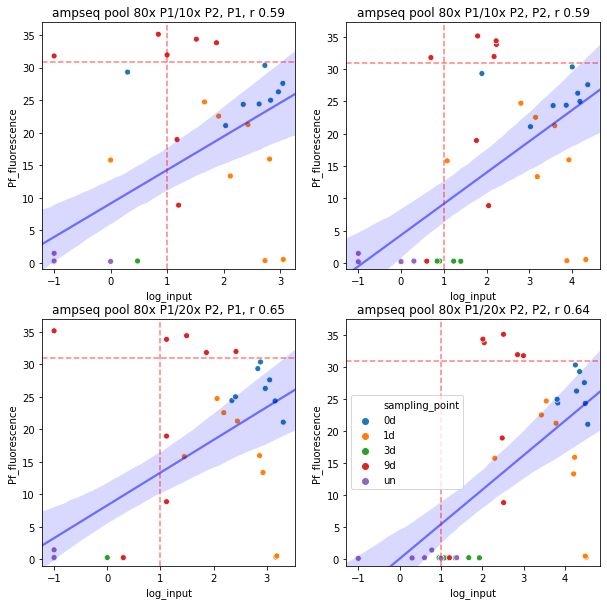


**Figure S15.** qPCR validation for lab-infected mosquitoes, experiment 1. Amplicon sequencing total read counts (log10) and qPCR fluorescence for 80X P1 and 10x or 20x P2 on the first set of lab-fed mosquito samples (8 samples per point). Vertical line - 10 reads cutoff used in DADA2 pipeline. Horizontal line - 5 pg/µl *P. falciparum* DNA (about 200 parasites/µl).


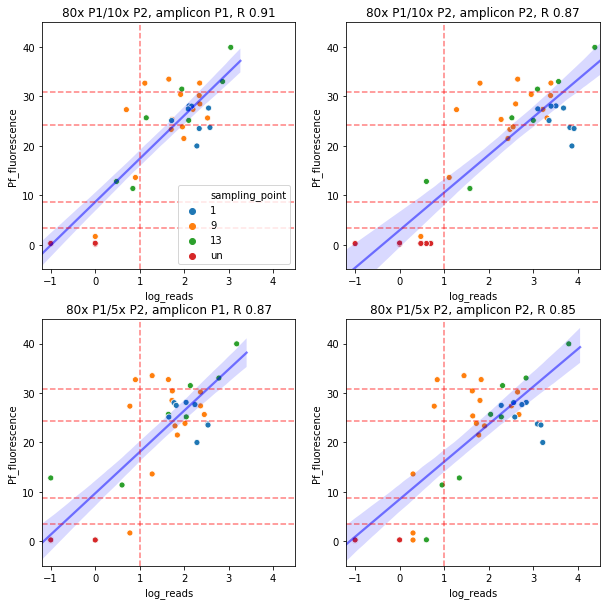


**Figure S16.** qPCR validation for lab-infected mosquitoes, experiment 2. Amplicon sequencing read counts and qPCR fluorescence for 80x P1 and 10x or 5x P2 on for a subset of second batch of lab-fed mosquito samples (e1_f16_9d - 16 samples, e2_f15_13d - 8 samples, e2_f15_1d - 8 samples, e2_f15_un - 8 samples). Vertical line - 10 reads cutoff used in DADA2 pipeline. Horizontal lines: fluorescence for 5 pg/µl, 500 fg/µl Pf, 50 fg/µl, and 5 fg/µl *P. falciparum* DNA (about 200, 20, 2 and 0.2 parasites/µl).

## Molecular Species ID validation using COI and ITS2 single marker Sanger sequencing

We compared our amplicon based approach for species determination of African and Southeast Asian samples to the most commonly used single marker Sanger sequencing approach using the nuclear internal transcribed spacer (ITS2) and mitochondrial cytochrome oxidase c subunit 1 (COI) PCR products.

The ITS2 region was PCR amplified using the ITS2A (5′-TGTGAACTGCAGGACACAT-3′) and ITS2B (5′-TATGCTTAAATTCAGGGGGT-3′) primers [(Beebe & Saul, 1995)](https://paperpile.com/c/WKXs3W/SYMh). Each 10 µl reaction consisted of 1x GoTaq® Green Master Mix, 1 µM ITS2A, 1 µM ITS2B, and 1 µl of DNA template. PCR cycling conditions were: 94°C for 5 min (enzyme activation); 25 cycles of 94°C for 30 sec (denaturation) - 52°C for 30 sec (annealing) - 72ºC for 1 min (extension); 72°C for 5 min (final extension); 10°C hold. After PCR, 1 µl of the reaction was run on 2% agarose gels to check for successful amplification (for different species product size varied from 450 bp to over 1,013 bp, the ladder’s upper limit).

The COI region was PCR amplified using the LCO1490 (5′-GGTCAACAAATCATAAAGATATTGG-3′) and HCO2198 (5′-TAAACTTCAGGGTGACCAAAAAATCA-3′) primers [(Folmer, Black, Hoeh, Lutz, & Vrijenhoek, 1994)](https://paperpile.com/c/WKXs3W/2lW9). Each 10 µl reaction consisted of 1x GoTaq® Green Master Mix, 1 µM LCO1490, 1 µM HCO2198, and 1 µl of DNA template. PCR cycling conditions were: 95°C for 5 min (enzyme activation); 5 cycles of 94°C for 40 sec (denaturation) - 45°C for 1 min (annealing) - 72ºC for 1 min (extension); 35 cycles of 94°C for 40 sec (denaturation) - 51°C for 1 min (annealing) - 72ºC for 1 min (extension); 72°C for 10 min (final extension); 10°C hold. After PCR, 1 µl of the reaction was run on 2% agarose gels to check for successful amplification (product size about 700 bp in all species).

PCR reactions were then purified and size selected using AMPure XP proprietary SPRI beads following the manufacturer’s protocol with minor modifications (1x bead to reaction volume ratio, washing performed twice using 180 µl of 80% ethanol, DNA eluted in 25 µl distilled water). Concentration was checked on a subset of samples using Nanodrop, and measured an average of 12±3 ng/µl for ITS2, and 18±6 ng/µl for COI. Samples that showed faint bands or multiple bands prior to purification were run again on a 2% agarose gel to check if spurious products were removed. Successful samples were then sent together with 10 µM primer dilutions (ITS2A, HCO2198) for Sanger sequencing using the Eurofins GATC SupremeRun 96 service (https://eurofinsgenomics.eu/en/custom-dna-sequencing/gatc-services/supremerun-plate/).

Sequencing data was successfully retrieved for 112 and 115 samples, for COI and ITS2 respectively. All Sanger sequences were checked and adjusted based on the original trace data in DNASTAR SeqMan Pro 15.2.0. Several methods were used to ascertain sample identity. We analysed BLAST 2.7.1 alignments against the NCBI nt database (Oct 20, 2019). Matches containing over 70% of the query sequence with identities higher than 96% for COI and 98% for ITS2 were considered to be the same species following (St Laurent et al. 2016). For COI sequences, we also performed a search in BOLD system version Jul-2020 (Ratnasingham and Hebert 2007). Finally, we generated alignments (with MAFFT v.7.407), counted SNP sites within each species, and reconstructed phylogenies (with FastTree v.2.1.10) for COI, ITS2, and pseudodiploid amplicon sequencing data representation. The resulting phylogenetic trees are available as Supplementary File 3. Species identification results are summarised in Supplementary File 2 tab “species identification”. Within-species diversity estimates are summarised in Supplementary File 2 tab “species summaries” and compared between amplicon sequencing and COI or ITS2 in Fig S17.


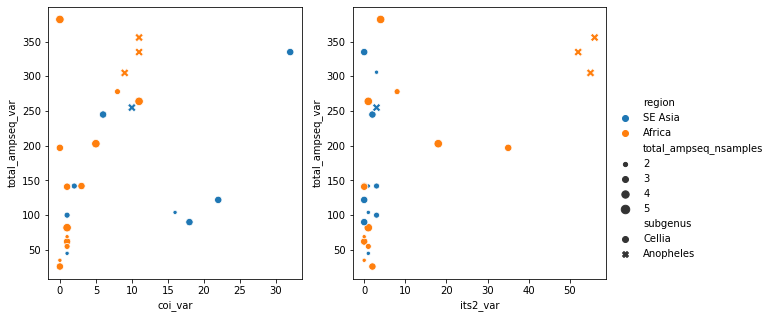


**Figure S17.** Within species variable sites for amplicon sequencing (y axis) and COI (x axis, left pane) or ITS2 (x axis, right pane). Colour - geographic region, marker size - number of samples in the analysis, shape - subgenus.

Species identities were generally in agreement between methods (Supplementary File 2 tab “species id comparison”). For many species, BOLD and NCBI GenBank database searches were successful in assigning species, species group or complex. However, accessions of closely related species were absent from the databases for the *Marshalli* group (*An. brohieri*, *An. hancocki* and the outgroup species *An. demeilloni*), basal Neomyzomiya species (*An.dureni* and *An. vinckei*), *An. gabonensis* and *An. rhodesiensis* (in our analyses both were found in Myzomyia series next to *An. funestus*, while previous studies suggest different phylogenetic positions). Using phylogenetic trees (Supplementary File 3), we evaluated the local relationships within and between species for COI, ITS2, and the amplicon panel. In many cases, species labels provided by partners corresponded to a single group of closely related samples on the tree suggesting those were true species calls. The exceptions were: 1) *An. brohieri, An. hancock*i (both from Marshallii group) and *An. demeilloni* that have variation consistent with a single species or species complex based on the panel, COI and ITS2; 2) *An. coustani, An. tenebrosus*, and *An. ziemannii* (all from Coustani group) that jointly formed a highly diverse clade with consistent substructure for all three markers - possibly a species complex or mislabeled species; 3) *An. paludis*, where one sample belonged to Coustani group and another was from a sister lineage; 4) samples labelled as *An. hyrcanus* and *An. nili* each consisted of two sister lineages according to all markers, suggesting greater diversity than currently recognized. We also found several samples that were obviously mis-labelled: two out of three *An. marshallii* and a single *An. sundaicus* sample. The signals for those were also consistent across all of COI, ITS2, and amplicon sequencing.

We evaluated within-species sequence diversity for the 27 species with at least 2 samples subject to amplicon sequencing, excluding *An. paludis*, *An. hyrcanus*, and *An. nili* that appear to each contain samples from highly diverged species. In amplicon sequencing, each species had an average of 8,943±665 bp sequence recovered with an average of 178±111 variable sites. For conventional markers, the recovered sequence length was 602±51 bp for COI and 537±143 bp for ITS2. For most species, 12 or fewer variable sites were observed across all samples. In several species, higher diversity was observed for a single marker. For COI, *An. rampae* had less than 96% sequence similarity and thus could be interpreted as different species, while *An. minimus, An. sundaicus*, and *An. vagus* had elevated variation still with over 96% similarity. For ITS2, all of *An. carnevalei, An. coustani, An. tenebrosus, An. theileri, An. ziemanni* had lower than 98% sequence similarity again suggesting those were different species (Supplementary File 2 tab “species summaries”, Fig S17). Based on comparison with other marker and amplicon sequencing results, we believe that those outliers indicate the problems with species identification approach based on a single marker gene.

## Sequence data processing pipelines design and benchmarking

For processing of the raw sequence data, two pipelines were implemented based on SeekDeep [(Hathaway, Parobek, Juliano, & Bailey, 2018)](https://paperpile.com/c/WKXs3W/zMqzb) and DADA2 [(Callahan et al., 2016)](https://paperpile.com/c/WKXs3W/R8ELt). SeekDeep was originally developed for multiplexed amplicon sequencing in the context of mixed *Plasmodium* infections and has a powerful method for detection of low frequency genotypes that relies on comparison of several replicates to remove spurious sequences introduced by PCR or sequencing errors. DADA2 is a tool that implements read denoising based on Illumina base qualities and haplotype merging. It was designed for metagenomics and does not have integrated demultiplexing steps, so we used cutadapt to split amplicon sequences within a sample. In both pipelines, the key outputs are the allelic sequences for sample-amplicon combinations and their associated read counts.

The pipeline based on SeekDeep v.2.6.4 was implemented in Snakemake v.5.5.3. The analysis is set up with SeekDeep setupTarAmpAnalysis command and then run with the generated runAnalysis.sh script. The pipeline was configured to use several replicates per sample when available. For all amplicons, read overlap statuses were set to “R1EndsInR2”, product length cutoff was set to a minimum of 150 and maxiumum of 299. The output files for resulting sequences and read counts were concatenated across amplicons and QC plots were generated with Python scripts.

The pipeline based on DADA2 v.1.10.0 was also implemented in Snakemake v.5.5.3. Replicate fastq files were split according to primer sequences using cutadapt v.2.5 with options “--no-indels --match-read-wildcards”. Primer matching and trimming was performed for forward and reverse reads independently, only read pairs matching both primers were taken in the analysis. Then haplotypes were inferred for each amplicon independently using the DADA2 pipeline recommended for ITS. This includes the filterAndTrim function with options “maxN=0, maxEE=c(2,2), truncQ=2, rm.phix=TRUE”. Resulting replicate haplotypes were merged into sample haplotypes and chimeric sequences were removed. Two filters were applied: at least 10 reads supporting sample-amplicon combination, at least 0.1 allele frequency for individual sample-amplicon alleles. Allelic sequences and read counts were summarized and QC plots were generated with a Python script.

For both pipelines, QC plots were focused on read counts, read filtering rates, allele counts, and allele imbalance. In our experience, these allow us to identify various problems with the input and processing. We ran the pipelines for each MiSeq run independently, which allows for better control of cross-contamination and sequencing biases.

We tested three versions of processing: DADA2, SeekDeep with three replicates per sample, and SeekDeep with one replicate per sample (replicate fastq files concatenated prior to analysis). Test dataset included a single Illumina MiSeq run consisting of 76 wild-caught mosquitoes from Africa, 11 lab-reared An. coluzzii, and 4 outgroup samples. The results suggest that SeekDeep performs well when given several replicates. However, more stringent filtering results in fewer recalled genotypes than DADA2. SeekDeep with a single replicate produces a lot of false positives manifesting as multiallelic sites (Table S3). Genotype prediction concordance was high for genotypes predicted by all methods. Similar observations on software performance were done in [(Early et al., 2019)](https://paperpile.com/c/WKXs3W/4CPpK). With all evidence taken together, and given the urge to use a single replicate per sample in the perspective, we chose to use DADA2 pipeline results for the downstream analysis.

**Table S3.** Pipeline performance comparison. Three pipeline versions tested: DADA2, SeekDeep with three or a single merged replicate. Recalled SA (sample-amplicon combinations) - SA with any sequence recovered (data recall), Multiallelic SA - SA with more than two alleles observed (excessive genotypes), Exclusive SA - SA observed only for this pipeline, Exclusive genotype - individual allele sequence observed only for this pipeline (both indicate potential false-positives).

|  | Recalled SA | Multiallelic SA | Exclusive SA | Exclusive genotypes |
| --- | --- | --- | --- | --- |
| DADA2 | 4045 | 65 | 220 | 423 |
| SeekDeep_x3 | 3241 | 11 | 0 | 9 |
| SeekDeep_x1 | 4069 | 861 | 160 | 1999 |

For the multispecies dataset comprising reference genomes and sequencing data for wild-caught mosquitoes from around the globe, we reconstructed species trees using data processed with a three-replicate SeekDeep approach (Fig S18) and DADA2 (Fig 3). Overall topologies were similar, but the SeekDeep-based tree supported the *Neomyzomyia* monophyly and its internal structure, e.g. monophyly of the *Ardensis* group consisting of the *Nili* complex, *An. dureni* and *An. vinckei*. Thus, more stringent data filtering by replicate comparison in SeekDeep looks beneficial for high-level phylogeny reconstruction.


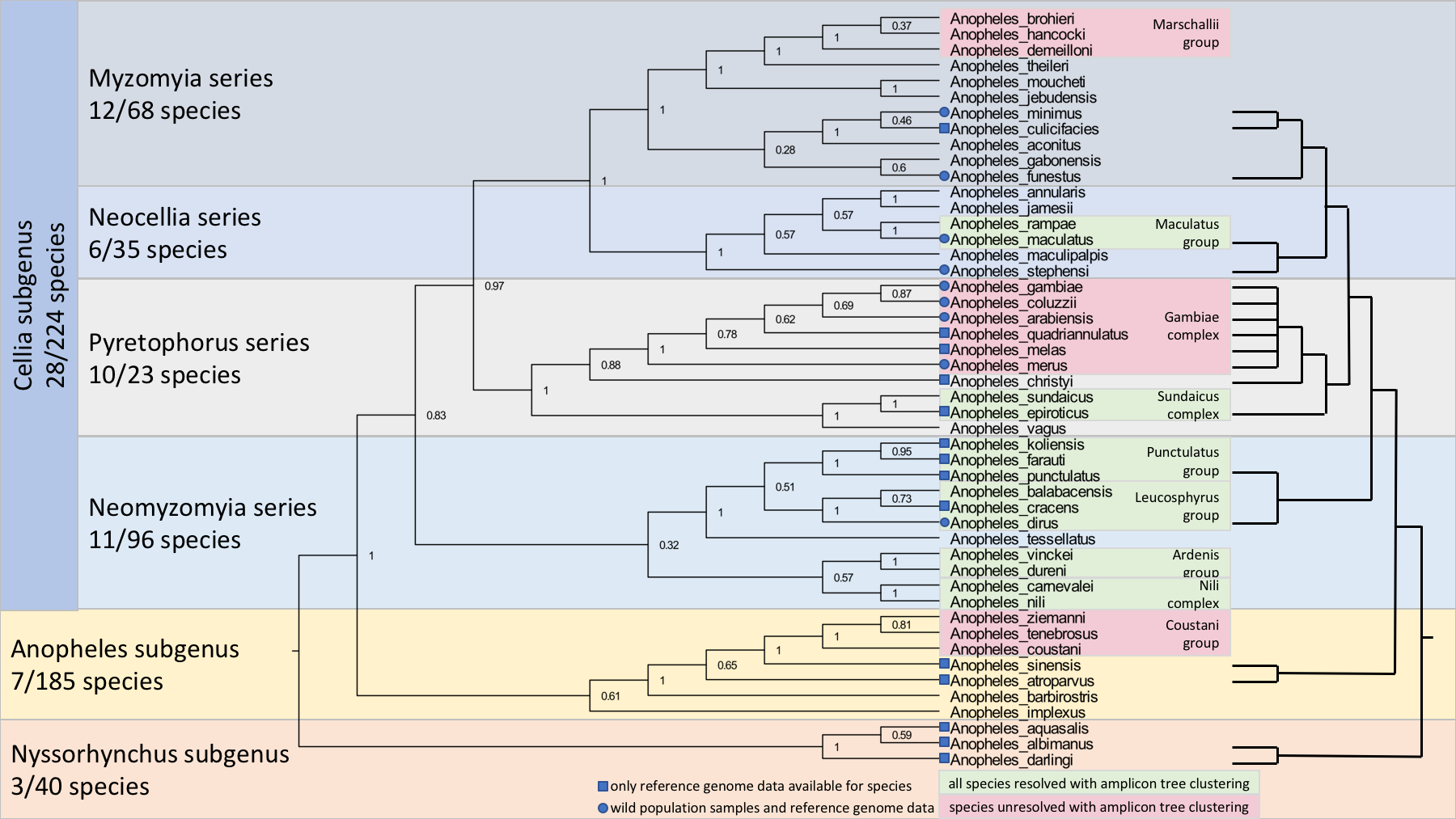


**Figure S18.** Left: species tree reconstructed from SeekDeep-processed amplicon sequencing data and reference genomes. Note the monophyly of *Neomyzomyia* series and *Ardensis* group. Right: whole-genome phylogeny from [(Neafsey et al., 2015)](https://paperpile.com/c/WKXs3W/GWicL).

## Panel applicability on outgroup species

The amplicon recovery rate was as low as 25% for the *Anopheles* species most distant to those groups we have the most sequence data for (Fig 2A), In order to test whether target sequences amplified outside the *Anopheles* genus, we generated sequence data for two *Culex sp*. samples, which belongs to the same family Culicidae, but to a different subfamily - Culicinae, rather than Anophelinae (diverged about 200 Mya [(Reidenbach et al., 2009)](https://paperpile.com/c/WKXs3W/0RCb2)). For these samples we obtained sequences for 9 and 14 amplicons (5 amplicons in common - 17, 27, 29, 52, 53), and the phylogenetic signal was sufficient to assign these samples as an outgroup to all *Anopheles* samples. We also tested two *Drosophila melanogaster* samples (sharing only order Diptera - ca 260 Mya divergence [(Logue et al., 2013)](https://paperpile.com/c/WKXs3W/81lKY)), for which only a single amplicon number 1 could be amplified. This corresponds to the exon of a highly conserved gene (AGAP005134, F-type H+-transporting ATPase subunit alpha) and could be examined more widely as a marker for exploring Diptera phylogeny.

## Distance-based species attribution

The dataset used here included mosquitoes collected from Africa, South-East Asia and South America, as well as target sequences extracted from reference genomes - a total of 164 samples belonging to 58 species from 4 subgenera (*Cellia* - 119 samples, 42 species; *Anopheles* - 36 samples, 9 species; *Nyssorhynchus* - 4 samples, 4 species; *Kerteszia* - 2 samples, 2 species).

Unique sequences for each of the 62 mosquito amplicons were aligned using MAFFT v.7.407 [(Katoh, Misawa, Kuma, & Miyata, 2002)](https://paperpile.com/c/WKXs3W/bBF1O). Based on these alignments, we collected per-amplicon statistics (Fig 2) and estimated pairwise distances between all unique sequences for each amplicon using the BioPython v.1.74 DistanceCalculator “identity” distance metric, which accounts for both substitutions and indels. For most species, distributions of within-species distances across all samples and amplicons rarely exceeded 0.1. At the same time, numerous spuriously high distances were observed for several species. By removing one or two samples per species, we were able to achieve reasonable distance distributions. In the case of *An. marshallii*, all 3 samples were removed. We hypothesised that the 8 outlier samples could be mis-identified based on morphology and/or the use of a single molecular marker.

Next, we aimed to cluster the sequences for each amplicon. In an ideal case, each cluster would correspond to a single species. However, this is only achievable if the within-species distances are strictly smaller than any between-species distances. In this case, the clustering threshold can be set between maximum within-species and minimum between-species distance. This concept is known as the “barcoding gap” [(Mallo & Posada, 2016)](https://paperpile.com/c/WKXs3W/xX80Y) and is actively applied in COI-based species identification. Inspection of within- and between-species distances for the 62 mosquitoes showed that only some amplicons demonstrate the reliable barcoding gap (Fig S19).


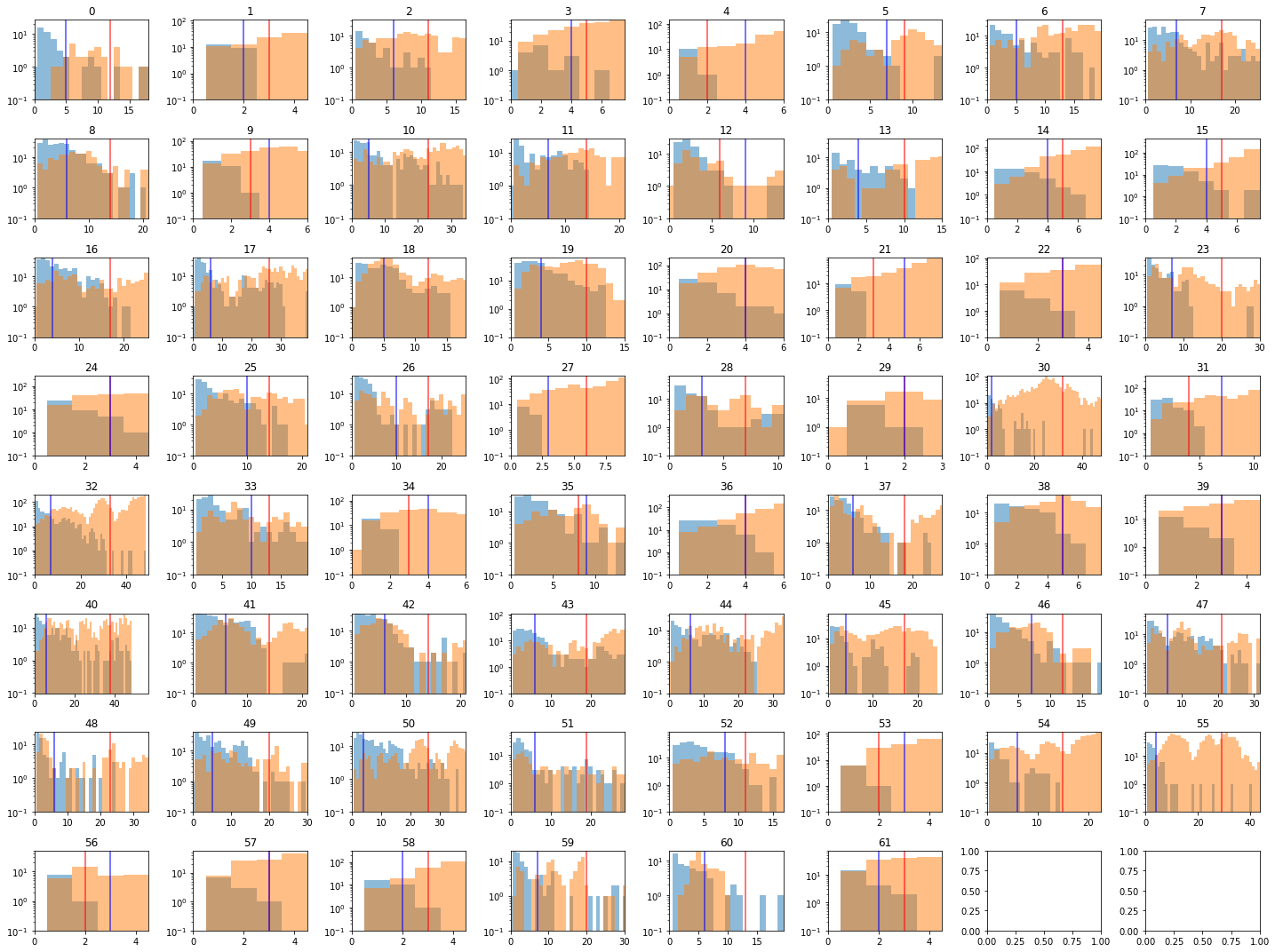


**Figure S19.** Barcodoing gap for 62 mosquito amplicons. Histograms of pairwise edit distances for sequenced samples and reference genomes - within (blue) and between (orange) species, higher distances excluded for better threshold visualisation. Vertical lines - maximum within-species diversity thresholds: based on sequencing data and reference genomes (“seq-ref”, red), based on Ag1000g Phase 2 (“ag1k”, blue).

Given that this barcoding gap was absent from some amplicons, we decided to use only within-species distances to inform the clustering threshold selection. For the dataset consisting of sequenced mosquitoes and reference genome extractions, we multiplied the “identity” distances to alignment length to get numbers of variants, and discarded the top 5% values to remove occasional outliers. Next, inverse numbers of variants were added to approach normal distribution. Clustering threshold (“seq-ref”) was estimated as rounded double standard deviation of the resulting distribution.

As an alternative source of maximum within-species variation estimates, we used SNP data from *An. gambiae* and *An. coluzzii* individuals sequenced as part of the Ag1000g phase 2 dataset [(“Ag1000G Phase 2 AR1 Data Release | MalariaGEN,” n.d.)](https://paperpile.com/c/WKXs3W/hFx5x). We extracted 1,142 (autosomal) or 1,058 (X chromosome) pairs of haplotypes using scikit-allel v.1.2.1 [(“Scikit-Allel - Explore and Analyse Genetic Variation — Scikit-Allel 1.2.1 Documentation,” n.d.)](https://paperpile.com/c/WKXs3W/0o0zN). For each amplicon, we set the clustering threshold (“ag1k”) as the maximum observed number of variants between two haplotypes within a species (*gambiae* or *coluzzii* - whichever was larger).

We performed two rounds of clustering with cd-hit-est v.4.8.1 [(Fu, Niu, Zhu, Wu, & Li, 2012)](https://paperpile.com/c/WKXs3W/7vT0o) using two sets of clustering thresholds - “seq-ref” and “ag1k” (vertical lines in Fig S18). These were used to set parameter “-c” - as the number of identical bases in alignment divided by the length of the shortest sequence. Word length (“-n”) was set to 10. Comparison of clustering results indicated better resolution at the species level when using the “ag1k” thresholds compared to using the “seq-ref” thresholds, while the amount of overly split clusters was similar (Fig S20). This could happen because Ag1000g samples represent continent-level population variation, while in the alignment dataset most species are represented by a few samples from a single location. Based on superior performance of “ag1k” thresholds, those were used in further analysis. Incorporation of wider population samples in the amplicon sequencing and estimation of the indel effects on the clustering algorithms performance are the prospective directions for future research.


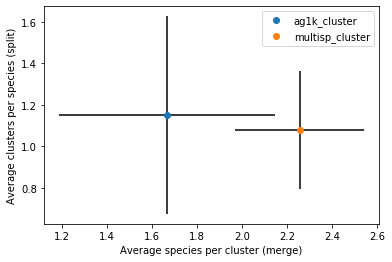


**Figure S20.** Sequence clustering performance using two sets of maximum within-species distance thresholds: derived from Ag1000g data (ag1k_cluster - 2 species, 100s of samples per species, no indels) and from alignment data (multisp_cluster - 34 species, 2-7 samples per species, with indels).

In order to predict species based on clustering results, we needed to establish a reference dataset. We included two types of data: all reference genome extractions and selected amplicon sequencing results. Filtering of sequencing results consisted of several steps. At sample level, we removed 8 outlier samples identified from within-species distance distributions as described above. Next, filtering was applied to individual sequences to exclude potential amplification or sequencing errors. First, outlier genotypes for sequenced samples were defined when species genotypes were split between several clusters and some clusters contained only a single genotype. Second, allelic genotype splits were defined if allelic sequences for the same sample-amplicon combination belonged to different clusters. As a result of all filtering procedures (8 outlier samples, 268 outlier genotypes, and 363 split alleles), out of 10,057 unique sequences observed in 135 sequenced samples and 28 reference genomes, 8,767 were retained in the reference dataset.

Sequences of the reference dataset were used to label clusters of sequences identified with “ag1k” similarity thresholds for each amplicon independently. Analysis of species co-occurrence in clusters indicated that groups of closely related species were often unresolved (Fig S21). Nevertheless, using the majority rule based on counts of cluster labels across all sample sequences proved to be a robust method for species prediction, where mismatches occured only for closely related species. To demonstrate the power of this approach in species identity prediction, for the samples excluded from the reference dataset, we predicted species identities using reference dataset for labelling (example in Fig 3B).


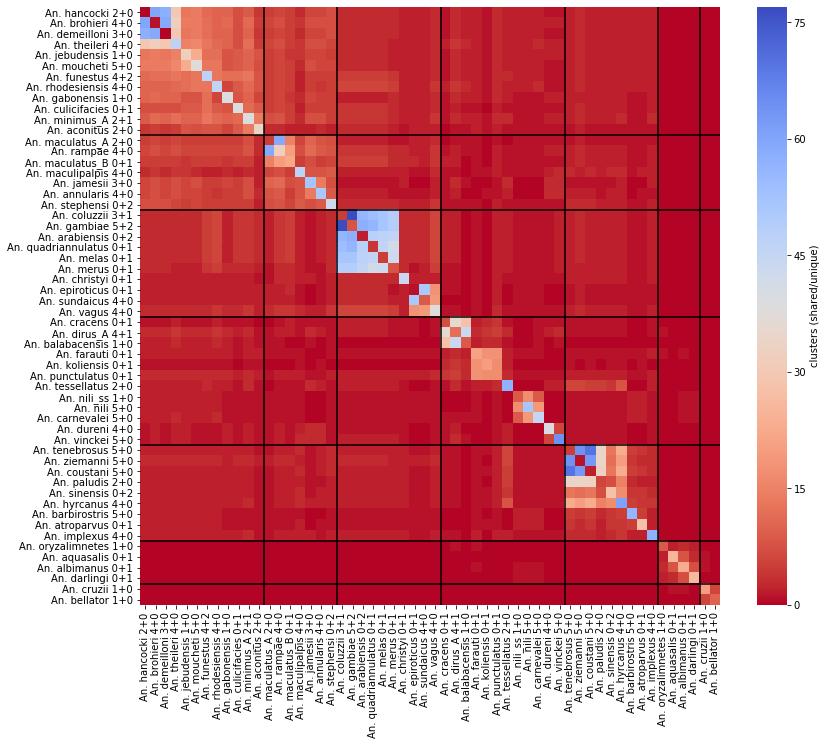


**Figure S21.** Species co-occurrence in sequence clusters (Ag1000g thresholds) summarized across 62 amplicons. Off-diagonal are pairwise species combinations in clusters, on diagonal are clusters consisting of only a given species. Black grid separates lineages: four series of *Celia* (*Myzomyia*, *Neocellia*, *Pyretophorus*, *Neomyzomyia*), *Anoheles*, *Nyssorhychus*, and *Kerteszia* subgenera.

# References

[Ag1000G phase 2 AR1 data release | MalariaGEN. (n.d.). Retrieved April 15, 2020, from](http://paperpile.com/b/WKXs3W/hFx5x) <https://www.malariagen.net/data/ag1000g-phase-2-ar1>

[Bass, C., Nikou, D., Blagborough, A. M., Vontas, J., Sinden, R. E., Williamson, M. S., & Field, L. M. (2008). PCR-based detection of Plasmodium in Anopheles mosquitoes: a comparison of a new high-throughput assay with existing methods. *Malaria Journal*, *7*, 177.](http://paperpile.com/b/WKXs3W/sJT4)

[Beebe, N. W., & Saul, A. (1995). Discrimination of all members of the Anopheles punctulatus complex by polymerase chain reaction--restriction fragment length polymorphism analysis. *The American Journal of Tropical Medicine and Hygiene*, *53*(5), 478–481.](http://paperpile.com/b/WKXs3W/SYMh)

[Callahan, B. J., McMurdie, P. J., Rosen, M. J., Han, A. W., Johnson, A. J. A., & Holmes, S. P. (2016). DADA2: High-resolution sample inference from Illumina amplicon data. *Nature Methods*, *13*(7), 581–583.](http://paperpile.com/b/WKXs3W/R8ELt)

[Cohuet, A., Simard, F., Toto, J.-C., Kengne, P., Coetzee, M., & Fontenille, D. (2003). Species identification within the Anopheles funestus group of malaria vectors in Cameroon and evidence for a new species. *The American Journal of Tropical Medicine and Hygiene*, *69*(2), 200–205.](http://paperpile.com/b/WKXs3W/zGTZ)

[Djouaka, R., Akoton, R., Tchigossou, G. M., Atoyebi, S. M., Irving, H., Kusimo, M. O., … Wondji, C. S. (2016). Mapping the distribution of *Anopheles funestus* across Benin highlights a sharp contrast of susceptibility to insecticides and infection rate to *Plasmodium* between southern and northern populations. *Wellcome Open Research*, *1*, 28.](http://paperpile.com/b/WKXs3W/ovCM)

[Early, A. M., Daniels, R. F., Farrell, T. M., Grimsby, J., Volkman, S. K., Wirth, D. F., … Neafsey, D. E. (2019). Detection of low-density Plasmodium falciparum infections using amplicon deep sequencing. *Malaria Journal*, *18*(1), 219.](http://paperpile.com/b/WKXs3W/4CPpK)

[Folmer, O., Black, M., Hoeh, W., Lutz, R., & Vrijenhoek, R. (1994). DNA primers for amplification of mitochondrial cytochrome c oxidase subunit 1 from diverse metazoan invertebrates. *Molecular Marine Biology and Biotechnology*, (5). Retrieved from](http://paperpile.com/b/WKXs3W/2lW9) <http://www.vliz.be/en/catalogue?module=ref&refid=64543>

[Fu, L., Niu, B., Zhu, Z., Wu, S., & Li, W. (2012). CD-HIT: accelerated for clustering the next-generation sequencing data. *Bioinformatics* , *28*(23), 3150–3152.](http://paperpile.com/b/WKXs3W/7vT0o)

[Gutaker, R. M., Reiter, E., Furtwängler, A., Schuenemann, V. J., & Burbano, H. A. (2017). Extraction of ultrashort DNA molecules from herbarium specimens. *BioTechniques*, *62*(2), 76–79.](http://paperpile.com/b/WKXs3W/d9nSP)

[Hathaway, N. J., Parobek, C. M., Juliano, J. J., & Bailey, J. A. (2018). SeekDeep: single-base resolution de novo clustering for amplicon deep sequencing. *Nucleic Acids Research*, *46*(4), e21.](http://paperpile.com/b/WKXs3W/zMqzb)

[Ibrahim, S. S., Mukhtar, M. M., Irving, H., Riveron, J. M., Fadel, A. N., Tchapga, W., … Wondji, C. S. (2020). Exploring the Mechanisms of Multiple Insecticide Resistance in a Highly Plasmodium-Infected Malaria Vector Anopheles funestus Sensu Stricto from Sahel of Northern Nigeria. *Genes*, *11*(4). doi:](http://paperpile.com/b/WKXs3W/W13rM) [10.3390/genes11040454](http://dx.doi.org/10.3390/genes11040454)

[Katoh, K., Misawa, K., Kuma, K.-I., & Miyata, T. (2002). MAFFT: a novel method for rapid multiple sequence alignment based on fast Fourier transform. *Nucleic Acids Research*, *30*(14), 3059–3066.](http://paperpile.com/b/WKXs3W/bBF1O)

[Koekemoer, L. L., Kamau, L., Hunt, R. H., & Coetzee, M. (2002). A cocktail polymerase chain reaction assay to identify members of the Anopheles funestus (Diptera: Culicidae) group. *The American Journal of Tropical Medicine and Hygiene*, *66*(6), 804–811.](http://paperpile.com/b/WKXs3W/jmEg)

[Liu, Q., & Sommer, S. S. (1998). Subcycling-PCR for multiplex long-distance amplification of regions with high and low GC content: application to the inversion hotspot in the factor VIII gene. *BioTechniques*, *25*(6), 1022–1028.](http://paperpile.com/b/WKXs3W/MkOt)

[Logue, K., Chan, E. R., Phipps, T., Small, S. T., Reimer, L., Henry-Halldin, C., … Serre, D. (2013). Mitochondrial genome sequences reveal deep divergences among Anopheles punctulatus sibling species in Papua New Guinea. *Malaria Journal*, *12*, 64.](http://paperpile.com/b/WKXs3W/81lKY)

[Mallo, D., & Posada, D. (2016). Multilocus inference of species trees and DNA barcoding. *Philosophical Transactions of the Royal Society of London. Series B, Biological Sciences*, *371*(1702). doi:](http://paperpile.com/b/WKXs3W/xX80Y) [10.1098/rstb.2015.0335](http://dx.doi.org/10.1098/rstb.2015.0335)

[Menze, B. D., Wondji, M. J., Tchapga, W., Tchoupo, M., Riveron, J. M., & Wondji, C. S. (2018). Bionomics and insecticides resistance profiling of malaria vectors at a selected site for experimental hut trials in central Cameroon. *Malaria Journal*, *17*(1), 317.](http://paperpile.com/b/WKXs3W/LEt15)

[Neafsey, D. E., Waterhouse, R. M., Abai, M. R., Aganezov, S. S., Alekseyev, M. A., Allen, J. E., … Besansky, N. J. (2015). Highly evolvable malaria vectors: The genomes of 16 Anopheles mosquitoes. *Science*, *347*(6217), 1258522.](http://paperpile.com/b/WKXs3W/GWicL)

[Nguyen-Dumont, T., Pope, B. J., Hammet, F., Mahmoodi, M., Tsimiklis, H., Southey, M. C., & Park, D. J. (2013). Cross-platform compatibility of Hi-Plex, a streamlined approach for targeted massively parallel sequencing. *Analytical Biochemistry*, *442*(2), 127–129.](http://paperpile.com/b/WKXs3W/8EX7y)

[Reidenbach, K. R., Cook, S., Bertone, M. A., Harbach, R. E., Wiegmann, B. M., & Besansky, N. J. (2009). Phylogenetic analysis and temporal diversification of mosquitoes (Diptera: Culicidae) based on nuclear genes and morphology. *BMC Evolutionary Biology*, *9*, 298.](http://paperpile.com/b/WKXs3W/0RCb2)

[Riveron, J. M., Huijben, S., Tchapga, W., Tchouakui, M., Wondji, M. J., Tchoupo, M., … Wondji, C. S. (2019). Escalation of Pyrethroid Resistance in the Malaria Vector Anopheles funestus Induces a Loss of Efficacy of Piperonyl Butoxide-Based Insecticide-Treated Nets in Mozambique. *The Journal of Infectious Diseases*, *220*(3), 467–475.](http://paperpile.com/b/WKXs3W/Dfl1I)

[Santos, D., Ribeiro, G. C., Cabral, A. D., & Sperança, M. A. (2018). A non-destructive enzymatic method to extract DNA from arthropod specimens: Implications for morphological and molecular studies. *PloS One*, *13*(2), e0192200.](http://paperpile.com/b/WKXs3W/84jgO)

[scikit-allel - Explore and analyse genetic variation — scikit-allel 1.2.1 documentation. (n.d.). Retrieved May 6, 2020, from](http://paperpile.com/b/WKXs3W/0o0zN) <https://scikit-allel.readthedocs.io/en/stable/>

[Scott, J. A., Brogdon, W. G., & Collins, F. H. (1993). Identification of single specimens of the Anopheles gambiae complex by the polymerase chain reaction. *The American Journal of Tropical Medicine and Hygiene*, *49*(4), 520–529.](http://paperpile.com/b/WKXs3W/6GlR)
